# Focal Adhesion Kinase (FAK)-Hippo/YAP transduction signaling mediates the stimulatory effects exerted by S100A8/A9-RAGE system in triple-negative breast cancer (TNBC)

**DOI:** 10.1186/s13046-022-02396-0

**Published:** 2022-06-03

**Authors:** Damiano Cosimo Rigiracciolo, Nijiro Nohata, Rosamaria Lappano, Francesca Cirillo, Marianna Talia, Sendi Rafael Adame-Garcia, Nadia Arang, Simone Lubrano, Ernestina Marianna De Francesco, Antonino Belfiore, J. Silvio Gutkind, Marcello Maggiolini

**Affiliations:** 1 Department of Pharmacy, Health and Nutritional Sciences, University of Calabria, 87036 Rende, Italy; 2 Moores Cancer Center, University of California, San Diego, La Jolla, CA USA; 3 MSD K.K., Tokyo, 102-8667 Japan; 4 Department of Clinical and Experimental Medicine, University of Catania, Garibaldi-Nesima Hospital, Catania, Italy; 5 Department of Pharmacology, University of California, San Diego, La Jolla, CA USA

**Keywords:** TNBC, MDA-MB-231, BT-549, S100A8/A9, RAGE, FAK, Hippo/YAP

## Abstract

**Background:**

Understanding the intricate signaling network involved in triple-negative breast cancer (TNBC) represents a challenge for developing novel therapeutic approaches. Here, we aim to provide novel mechanistic insights on the function of the S100A8/A9-RAGE system in TNBC.

**Methods:**

TNM plot analyzer, Kaplan-Meier plotter, Meta-analysis, GEPIA2 and GOBO publicly available datasets were used to evaluate the clinical significance of S100A8/A9 and expression levels of S100A8/A9, RAGE and Filamin family members in breast cancer (BC) subtypes. METABRIC database and Cox proportional hazard model defined the clinical impact of high RAGE expression in BC patients. Multiple bioinformatics programs identified the main enriched pathways within high RAGE expression BC cohorts. By lentiviral system, TNBC cells were engineered to overexpress RAGE. Western blotting, immunofluorescence, nucleus/cytoplasm fractionation, qRT-PCR, gene silencing and luciferase experiments were performed to identify signal transduction mediators engaged by RAGE upon stimulation with S100A8/A9 in TNBC cells. Proliferation, colony formation and transwell migration assays were carried out to evaluate the growth and migratory capacity of TNBC cells. Statistical analysis was performed by ANOVA and independent t-tests.

**Results:**

We found a remarkable high expression of S100A8 and S100A9 in BC, particularly in HER2-positive and TNBC, with the latter associated to worst clinical outcomes. In addition, high RAGE expression correlated with a poor overall survival in BC. Next, we determined that the S100A8/A9-RAGE system triggers FAK activation by engaging a cytoskeleton mechanosensing complex in TNBC cells. Through bioinformatics analysis, we identified the Hippo pathway as the most enriched in BC patients expressing high RAGE levels. In accordance with these data, we demonstrated the involvement of S100A8/A9-RAGE-FAK signaling in the control of Hippo/YAP activities, and we established the crucial contribution of RAGE-FAK-YAP circuitry in the growth and migratory effects initiated by S100A8/A9 in TNBC cells.

**Conclusions:**

The present study provides novel mechanistic insights on RAGE actions in TNBC. Moreover, our findings suggest that RAGE-FAK-YAP transduction pathway could be exploited as a druggable system halting the aggressive TNBC subtype.

**Supplementary Information:**

The online version contains supplementary material available at 10.1186/s13046-022-02396-0.

## Background

Breast cancer (BC) has widely exceeded lung and colorectal cancer as the most frequently diagnosed women malignancy in the United States during the first months of 2022 [1]. Generally, BC subtypes are classified by the expression of estrogen receptor (ER), progesterone receptor (PR) and human epidermal growth factor receptor-2 (HER-2) [2]. In this context, triple-negative breast cancer (TNBC), coined under this term due to the lack of ER, PR and HER-2 expression, is considered the breast malignant subtype associated with the least favorable therapeutic options and clinical outcomes [3]. Although chemotherapy remains the backbone of treatment for TNBC patients, the use of neo-adjuvant chemotherapy regimens in combination with immune checkpoint inhibitors (ICIs) has shown promising responses in those patients with early-stage TNBC [4–6]. Therefore, the identification of novel biomarkers as suitable therapeutic targets for metastatic TNBCs could facilitate combinatorial personalized treatments as well as better clinical outcomes.

Among the potential prognostic biomarkers for TNBC, increasing attention has been recently center around the role of S100 proteins, a large family of calcium-binding cytosolic proteins tightly linked to inflammatory disorders, neurological pathologies and even progression of diverse tumors, including BC [7, 8]. Within the S100-Ca^2+^ binding protein family, elevated levels of S100A8 and S100A9 have been detected in ER-negative and TNBC patient cohorts [9, 10]. Of clinical relevance, high percentage of infiltrating S100A8+ myeloid cells as well as S100A9 expression have been proposed to represent a potential mechanism driving worse prognosis in BC, mostly in TNBC basal-like subtypes [10, 11]. Mechanistically, S100A8 and S100A9 act as a hetero-complex damage-associated molecular pattern (DAMP), namely calprotectin, by binding cell surface proteins like the immunoglobulin Receptor for Advanced Glycation End-products (RAGE), thereby initiating the modulation of a wide range of pro-inflammatory responses and oncogenic signaling [12–14]. Of note, RAGE has been suggested as a suitable candidate biomarker for TNBC diagnosis on the basis of its up-regulation and association with a poor prognosis in invasive and metastatic TNBCs [15]. In addition, RAGE has been shown to drive cell invasiveness and metastasis in different TNBC models by regulating both tumor-intrinsic and extrinsic cell effects [16]. In accordance with these findings, these processes were halted by targeting RAGE [16]. In addition, S100A8/A9 binding to RAGE promoted epithelial-mesenchymal transition (EMT) in TNBC cells and lung metastasis in a TNBC xenograft mouse model [17]. To date, pharmacological agents targeting RAGE tested in clinical trials have shown promising results for the treatment of certain disorders, such as cardiovascular-associated comorbidities, acute respiratory distress syndrome, and early stage of Alzheimer’s disease [18, 19]. However, it remains to be assessed whether the tested drugs might be considered as potential anti-cancer therapeutics agents, including aggressive TNBCs.

By decoding the oncogenic signaling circuitries involved in TNBC progression, emerging studies, including ours, have highlighted the role of the non-receptor focal adhesion kinase (FAK) and the Hippo pathway component Yes-associated protein (YAP) [20–24] in TNBC progression. Although fibroblast transition to a cancer-associated fibroblast (CAF)-like phenotype was inhibited by targeting both RAGE and integrin-mediated mechano-transduction that in turn resulted in the reduction of FAK activation and YAP nuclear localization [25], the functional connection of RAGE with FAK/Hippo/YAP pathway in TNBC is still poorly understood.

Here, we show that S100A8/A9 activation of RAGE leads to actomyosin-mediated FAK activation, YAP nuclear accumulation and gene regulation in TNBC cells. Notably, both proliferative and migratory abilities of TNBC cells were suppressed by targeting the S100A8/A9-RAGE-FAK-YAP circuitry. Our novel findings contribute to a better understanding of the molecular mechanisms initiated by RAGE in TNBC progression, suggesting the potential usefulness of agents targeting RAGE, as single agents or in combination treatments, in order to halt this highly metastatic breast malignancy.

## Materials and methods

### Bioinformatics analysis

Gene chip data expression analysis of S100A8 and S100A9 was performed using TNMplot differential gene expression analysis in Tumor, Normal and Metastatic tissues analyzer (https://tnmplot.com/analysis) [26]. *p-value* was determined by Mann_Whitney test. The TIMER2.0 database was used for S100A8 and S100A9 gene expression correlation in TNBC samples by selecting Spearman analysis methodology. Overall Survival (OS) and Relapse Free Survival (RFS) for S100A8 and S100A9 in breast cancer were generated by Kaplan-Meier Plotter for Breast Cancer (https://kmplot.com/analysis/index.php?p=service&cancer=breast) [27]. Differential gene expression levels of S100A8 and S100A9 between normal, TNBC, HER2-positive, Luminal-A and Luminal-B BC subtypes were collected from Gene Expression Profiling Interactive Analysis 2 (GEPIA2) database (http://gepia2.cancer-pku.cn/#index) (*p-value* Cutoff was 0.01). GOBO gene expression-based outcome for breast cancer online tool provided by the Lund University (https://co.bmc.lu.se/gobo/gsa.pl) was used to further explore S100A8 and S100A9 expression in BC subtypes as well as the combined S100A8/A9 expression among all BC subgroups [28]. Gene expression data in breast cancer were also obtained from Molecular Taxonomy of Breast Cancer International Consortium (METABRIC) in cBioPortal database (https://www.cbioportal.org/) [29]. The mRNA expression Z-scores and information on the clinical samples corresponding to patients with BC were collected from cBioPortal. To categorize genes into molecular pathways based on gene set enrichment analysis (GSEA) [30] or KEGG pathway analysis [31], we employed the WEB-based Gene SeT AnaLysis Toolkit (WebGestalt) (http://www.webgestalt.org/) [32] and Enrichr (https://maayanlab.cloud/Enrichr/) [33] programs, respectively. For the Kaplan–Meier survival analysis and multivariate Cox proportional hazards regression analysis, we divided METABRIC data into low and high groups for the expression of RAGE based on the cut-off of *Z-score* of 1. We analyzed the OS time between the groups by log-rank test. In addition, multivariate statistical technique was performed using Cox proportional hazards model. Statistical analyses were performed using GraphPad Prism 7 (GraphPad Software, La Jolla, CA, USA) and JMP Pro 15 (SAS Institute Inc., Cary, NC, USA). Volcano plot was generated by Appyters [34], whereas Venn Diagram was prepared by Canva program (https://www.canva.com).

### Meta-analysis

We searched the public database PROGgeneV2, which collects data in association with cohort studies from public repositories such as TCGA, GEO, and EBI Array Express [35]. Microarray or RNA-sequencing data with overall survival information for S100A8, S100A9, RAGE and FLNA in breast cancer are available online at PROGgeneV2 (http://www.progtools.net/gene/index.php). Data were collected on April 10, 2022 and combined by means of common effect model and random effects model based on the hazard ratios and their upper 95% confidence intervals. The meta-analysis was performed using EZR software [36].

### S100A8/A9-RAGE proposed binding interaction

The model for the potential interaction surface in the RAGE - S100A8/A9 complex was prepared using the previous resolved structures for RAGE ectodomains (PDB 4P2Y) and RAGE V-domain in complex with S100A6 (PDB 2M1K). The structures were examined and modelled with UCSF Chimera, developed by the Resource for Biocomputing, Visualization, and Informatics at the University of California, San Francisco [37–39].

### Cell culture

HEK293T and MDA-MB-231 TNBC cells were purchased from ATCC (Manassas, VA) and cultured in High Glucose DMEM (D-6429, Sigma-Aldrich Inc., St. Louis, MO) supplemented with 10% FBS (Sigma-Aldrich Inc., St. Louis, MO), 1× antibiotic/antimycotic solution (Sigma-Aldrich Inc., MO) and 5 μg/ml plasmocin™ prophylactic (InvivoGen, CA). BT-549 TNBC cells were kindly provided by Dr. Y. Wang (Moores Cancer Center, University of California San Diego, UCSD) and cultured in RPMI-1640 (R-8758, Sigma-Aldrich Inc., St. Louis, MO) media supplemented with 10% FBS (Sigma-Aldrich Inc., St. Louis, MO), 1× antibiotic/antimycotic solution (Sigma-Aldrich Inc., MO) and 5 μg/ml plasmocin™ prophylactic (InvivoGen, CA). Cells were grown in a 37 °C incubator with 5% CO_2_, used less than 6 months after thawing and proved to be Mycoplasma free by using the MycoAlert PLUS Mycoplasma Detection Kit (Lonza) according to the ATCC suggestions. Cells to be processed for immunoblot, immunofluorescence, and luciferase, and RT-PCR experiments were switched to medium without serum the day before the indicated treatments.

### Reagents and drugs

Recombinant Human S100A8/A9 Heterodimer (carrier-free) (#753406) was purchased from Biolegend® (San Diego, CA). RAGE antagonist FPS-ZM1 (#S8185), AKT inhibitor Ipatasertib (#S2808), ROCK inhibitor Y-27632 2HCl (#S1049) and Myosin II inhibitor Blebbistatin (#S7099) were obtained from Selleckchem. FAK inhibitor VS-4718 (# HY-13917) and YAP/TEAD binding disruptor Verteporfin (# HY-B0146) were purchased from MedChemExpress (MCE). rhS100A8/A9 was diluted at 100 μg/mL in 1X PBS, while each pharmacological inhibitor was prepared as a 10 mM stock solution in DMSO.

### Cloning, lentivirus production and generation of TNBC cells stable overexpressing RAGE

pcDNA3-RAGE (Addgene Plasmid #71435) vector was cloned into the lentiviral expression vector pLESIP, obtained from dr. J.S. Gutkind lab (Moores Cancer Center, University of California San Diego, UCSD). Briefly, pLESIP backbone was digested overnight at 37 °C by using NheI-HF® (#R3131S) and EcoRI-HF® (#R3101S) restriction enzymes in the presence of 10X CutSmart® Buffer (New England Biolabs, NEB). A specific nucleotide sequence sticking between NheI and EcoRI sites within the pcDNA3-RAGE vector was amplified by qPCR. Primers were as follows: F: 5′-CATGCTAGCGCCACCATGGCAGCCGGAACAGCAGTT -3′, R: ATGGAATTCTCAAGGCCCTCCAGTACTAC-3′. pcDNA3-RAGE qPCR amplified product was purified and subsequently digested with NheI-HF® (#R3131S, New England Biolabs, NEB) and EcoRI-HF® (#R3101S, New England Biolabs, NEB) restriction enzymes in the presence of 10X rCutSmart® Buffer (#B6004S, New England Biolabs, NEB). Both products derived from pLESIP and pcDNA3-RAGE enzymatic digestion were incubated overnight with T4 DNA Ligase (#M0202S, New England Biolabs, NEB) in the presence of 10X T4 DNA Ligase Buffer (#B0202S, New England Biolabs, NEB) for cloning at 4 °C and then subjected to bacteria transformation. Ten individual colonies were picked, screened and a double enzymatic digestion process was performed to ascertain the success of RAGE cloning into the lentiviral expression vector pLESIP. For lentiviruses production, 10 cm dishes with 70% confluent HEK293T cells were transiently co-transfected using TurboFect™ Transfection Reagent (#R0533, ThermoFisher Scientific) with VSV-G (2 μg) and psPAX2 (4 μg) packaging plasmids, and pLESIP-EV (6 μg) or pLESIP-RAGE (6 μg) vectors, following the manufacturer’s protocol. Viral supernatants were collected at 48 and 72 hours after transfection, respectively. The supernatants were concentrated by ultracentrifugation at 15,000 g for 10 minutes. One day before the transduction, MDA-MB-231 and BT-549 TNBC cells were plated in 10 cm dishes. When cells reached 50% of confluence, polybrene (10 μg/ml) was mixed with concentrated lentiviruses and added to each plate. After 72 hours of incubation, the transduced TNBC cells were selected with puromycin at 1 μg/ml final concentration. TNBC cells were continuously maintained for 1 week at 1 μg/ml puromycin to avoid loss of RAGE DNA. RAGE overexpression in MDA-MB231 and BT-549 TNBC cells was ascertained through RT-PCR and western blotting assays.

### Western blotting assay and antibodies

MDA-MB-231 and BT-549 TNBC cells overexpressing RAGE were grown in 6-well plates. Forty-eight hours post-seeding, cell media was replaced with serum-free media and cells were exposed to the respective treatments or transfected with the respective siRNAs. Then, cells were harvested after rinsing twice with cold phosphate-buffered saline (PBS) and lysed in RIPA buffer containing 50 mM Tris-HCl, 150 mM NaCl, 1 mM EDTA, 1% NP-40 and supplemented with HaltTM Protease and Phosphatase Inhibitor Cocktail (#78440, ThermoFisher Scientific). Each lysate was sonicated 3 times for 5 seconds, incubated for 15 minutes on ice and centrifuged for 15 minutes at 4 °C. The concentration of supernatants was measured by using DC Protein Assay (#5000111, Bio-Rad). Equal amounts of protein (10 μg) were loaded for SDS-PAGE, and transferred to PVDF membranes. The membranes were blocked with 5% non-fat milk in TBS-T buffer for 1 hour at room temperature, washed in TBS-T buffer 3 times, and incubated overnight with primary antibodies diluted in 5% BSA in TBS-T buffer. The next day, after washing by TBS-T buffer 3 times, the membranes were incubated with secondary antibodies (HRP-conjugated goat anti-mouse or anti-rabbit IgG at 1:20,000 dilution, Southern Biothech) diluted by 5% non-fat milk in TBS-T buffer for 1 hour at room temperature. Immobilon Western Chemiluminescent HRP substrate (Millipore, MA) was used for protein’s detection. The following primary antibodies were used for immunoblot analysis: anti-RAGE (Clone A17158D, 1:1000) was purchased from Biolegend® (San Diego, CA). Anti-pY397FAK (#8556S, 1:1000), FAK (#3285S, 1:1000), pS473AKT (#4060, 1:1000), AKT (#9272, 1:2000), Rho-A (#2117, 1:2000), pS127YAP (#4911, 1:1000), YAP (#14074, 1:2000), pT183/180-MST1/2 (#49332, 1:1000), MST1 (#3682, 1:1000), CTGF (#86641, 1:1000), Cyr61 (#14479, 1:1000), and ꞵ-actin (#4967, 1:5000) were purchased from Cell Signaling Technology (MA). Lamin A/C (E-1, 1:2000) was obtained from Santa Cruz Biotechnology (Dallas, TX).

### Nuclear and cytoplasm extraction

Extraction of subcellular fractionated lysates was performed using The Nuclear & Cytoplasmic Extraction Kit (Cat. #786–182, G-Biosciences) following the manufacturer instructions. Briefly, after treatments, MDA-MB231 TNBC cells were harvested and centrifuged at 500 x g for 5 minutes. Then, pellet was washed with cold PBS, centrifuged at 500 x g for 5 minutes, and an appropriate volume of SubCell Buffer-1 was added. After resuspension of pellet, an appropriate volume of SubCell Lysis Reagent was added to each tube, appropriately vortexed and incubate for 1 minute on ice, and finally centrifuged for 5 minutes at maximum speed. The supernatant (cytosol fraction) was collected in new tubes, while the pellet was suspended with Nuclear Extraction Buffer, incubated for 30 minutes on ice and finally centrifuged for 10 minutes at maximum speed. The supernatant was collected in new tubes (nuclear fraction). The concentration of supernatants was measured by using DC Protein Assay (#5000111, Bio-Rad). Equal amounts of protein (20 μg) were loaded for SDS-PAGE and the protocol of western blotting assay (as described above) was executed. The following primary antibodies were used for immunoblot analysis: YAP (#14074, 1:2000), ꞵ-actin (#4967, 1:5000) and Lamin A/C (E-1, 1:2000).

### RNA extraction and real-time PCR

MDA-MB-231 TNBC cells overexpressing RAGE were grown in 6-well plates. Forty-eight hours post-seeding, cell media was replaced with serum-free media and cells were exposed to the respective treatments or transfected with the respective siRNAs. RNA was extracted using RNeasy Mini Kit following the manufacturer’s instruction (#74104, Qiagen, Hilden, Germany), spectrophotometrically quantified and 500 ng of total RNA was used for cDNA synthesis using SuperScript™ VILO™ cDNA Synthesis Kit (#11754250, Thermo Fisher Scientific, CO). Real-time PCR was performed using SYBR™ Select Master Mix (#4472908, Thermo Fisher Scientific, CO). Primer sequences were designed through PrimerBank PCR Primers for Gene Expression Detection and Quantification (https://pga.mgh.harvard.edu/primerbank/) [40]. The following primers were used: GAPDH F: 5′-GAGTCAACGGATTTGGTCGT-3′, R: TTGATTTTGGAGGGATCTCG-3′, CTGF F: 5′-GTTTGGCCCAGACCCAACTA-3′, R: GGCTCTGCTTCTCTAGCCTG-3′, Cyr61 F: 5′-CAGGACTGTGAAGATGCGGT-3′, R: GCCTGTAGAAGGGAAACGCT-3′, and FLN2 F: 5′- CTTATCGCGCTGTTGGAGGT -3′, R: 5′- GCCACCGACACGTTCTCAA − 3′.

### Knockdown by siRNA

MDA-MB-231 and BT-549 TNBC cells overexpressing RAGE were transfected with siRNAs using Lipofectamine RNAiMAX Reagent (Thermo Fisher Scientific) following the manufacturer’s protocol. The sequences of siRNA targeting RAGE (ON-TARGETplus Human AGER siRNA - SMARTpool, #L-003625-00-0005), FAK (ON-TARGETplus Human PTK2 siRNA - SMARTpool, #L-003164-00-0005), YAP (ON-TARGETplus Human YAP1 siRNA - SMARTpool, #L-012200-00-0005), Rho-A (ON-TARGETplus Human Rho-A siRNA - SMARTpool, #L-003860-00-0005), CTGF (ON-TARGETplus Human CTGF (1490) siRNA - Individual, #J-012633-10-0002) and FLNA (ON-TARGETplus Human FLNA (2316) siRNA - Individual, #J-012579-05-0002) were purchased from Horizon Discovery Biosciences Limited (CO), while siRNA negative control (SIC001) was obtained from Sigma-Aldrich, (MO).

### Luciferase assay

MDA-MB-231 TNBC cells overexpressing RAGE were plated into 24-well plates. Twenty-four hours post-seeding, cell media was replaced with serum-free media and cells were co-transfected with 5 ng/well of pCEFL-3x-Flag-Renilla-luciferase and 500 ng/well of 8xGTIIC-luciferase (Addgene Plasmid #34615). Transfection was performed by using TurboFect™ Transfection Reagent, following the manufacturer’s protocol (#R0533, ThermoFisher Scientific). After 12 hours post-transfection, cells were stimulated for 6 hours with rhS100A8/A9 (100ng/ml) alone or in combination with RAGE antagonist FPS-ZM1 (1 μM) and FAK inhibitor VS-4718 (1 μM). The detection of the luciferase activity was conducted using a Dual-Glo Luciferase Assay Kit (Promega, WI) and a Microtiter plate luminometer (Dynex Tech., VA).

### Y397FAK and FLNA immunofluorescence microscopy

MDA-MB-231 TNBC cells overexpressing RAGE (1 × 10^4^) were cultured on coverslips in regular growth medium for 24 hours. Then, cells were serum-deprived for 12 hours and stimulated for 30 minutes with rhS100A8/A9 (100ng/ml) used alone or in combination with the RAGE antagonist FPS-ZM1 (1 μM), FAK inhibitor VS-4718 (1 μM) and ROCK inhibitor Y-27632 (1 μM). Next, cells were washed with 1X PBS, fixed with 3.7% formaldehyde diluted in PBS for 10 minutes and permeabilized using 0.05% Triton X-100 for 15 minutes. Fixed cells were blocked with 3% BSA diluted in 1X PBS for 1 hour at room temperature, and incubated overnight at 4 °C with anti-pY397FAK (#8556S, 1:250, Cell Signaling Technology, MA). After incubation, cells were washed with 1X PBS and incubated with Alexa Fluor™ 488 goat anti-rabbit (1:500) (Invitrogen, CA) for 1 hour at room temperature. Cells were then washed with 1X PBS and incubated in PBS buffer containing 4′, 6-diamidino-2-phenylindole (DAPI) (Molecular Probes, OR) for 15 minutes at room temperature for nuclear staining. Lastly, coverslips were extensively washed with 1X PBS and mounted on Polysine® microscope slides (#P4981–001, ThermoFisher Scientific) covered with a small amount of ProLong™ Gold antifade reagent solution (Invitrogen, CA). Images showing focal adhesions and quantification of Y397FAK per cell (number of FAs/cell) were acquired and analyzed with an Axio Imager Z1 microscope equipped with ApoTome system controlled by ZEN 2012 software (Carl Zeiss, NY). The same protocol was applied for FLNA immunofluorescence staining. Here, MDA-MB231 TNBC cells overexpressing RAGE (1 × 10^4^) cultured on coverslips were treated for 6 hours with rhS100A8/A9 (100ng/ml) used alone or in combination with RAGE antagonist FPS-ZM1 (1 μM), FAK inhibitor VS-4718 (1 μM) and YAP/TEAD disruptor Verteporfin (1 μM). Fixed cells blocked with 3% BSA diluted in 1X PBS were incubated overnight at 4 °C with anti-FLNA (Clone 1G4H3, # 67133–1-Ig, Proteintech, IL) and the day after with Alexa FluorTM 546 goat anti-mouse (Invitrogen, CA), followed by the aforementioned procedure. Images showing cytoplasmic FLNA staining and FLNA fluorescence intensity were acquired and analyzed with an Axio Imager Z1 microscope equipped with ApoTome system controlled by ZEN 2012 software (Carl Zeiss, NY).

### YAP nuclear immunofluorescence staining

MDA-MB-231 TNBC cells overexpressing RAGE were cultured on 24-well plates in complete regular media until they reached 50–60% of confluence. Then, cells were serum-deprived for 12 hours and stimulated for 1 hour with rhS100A8/A9 (100ng/ml) used alone or in combination with the RAGE antagonist FPS-ZM1 (1 μM) and FAK inhibitor VS-4718 (1 μM). Next, cells were washed with 1X PBS, fixed with 3.7% formaldehyde diluted in 1X PBS for 10 minutes and permeabilized using 0.05% Triton X-100 for 15 minutes. Fixed cells were blocked with 3% BSA diluted in 1X PBS for 1 hour at room temperature, and incubated overnight at 4 °C with anti-YAP (#14074, 1:300, Cell Signaling Technology, MA). After incubation, cells were washed with 1X PBS and incubated with Alexa Fluor™ 488 goat anti-rabbit (1:500) (Invitrogen, CA) for 1 hour at room temperature. Cells were then washed with 1X PBS and incubated in PBS buffer containing 4′, 6-diamidino-2-phenylindole (DAPI) (Molecular Probes, OR) for 15 minutes at room temperature for nuclear staining. Lastly, cells were extensively washed with 1X PBS, and YAP nuclear accumulation pictures as well as quantification of nuclear YAP per cell were acquired and analyzed with an Axio Imager Z1 microscope equipped with ApoTome system controlled by ZEN 2012 software (Carl Zeiss, NY).

### Cell proliferation assay

MDA-MB-231 and BT-549 TNBC cells overexpressing RAGE (1 × 10^3^) were seeded in 24-well plates in regular growth medium, and after 24 hours cells were washed with 1X PBS, incubated in medium containing 2.5% charcoal-stripped FBS and treated with rhS100A8/A9 (100ng/ml) alone or in combination with the RAGE antagonist FPS-ZM1 (1 μM) and FAK inhibitor VS-4718 (1 μM). Media and treatments were renewed every day. The proliferation rate was calculated counting the cells after 72 hours using the Countess Automated Cell Counter, as recommended by the manufacturer’s protocol (ThermoFisher Scientific). For proliferation assay performed in the presence of siRNAs, BT-549 TNBC cells overexpressing RAGE were transfected with siRNA targeting Control (20 nM) and siRNA targeting CTGF (20 nM), respectively, and after 72 hours cells were trypsinized, counted and 1 × 10^3^ cells were seeded in 24-well plates in regular growth medium. After 24 hours cells were washed with 1X PBS, incubated in medium containing 2.5% charcoal-stripped FBS and then stimulated with rhS100A8/A9 (100ng/ml) for 72 hours, while remaining cells were processed to ascertain CTGF knockdown by RT-PCR. The proliferation rate was calculated counting the cells after 72 hours by using the Countess Automated Cell Counter, as recommended by the manufacturer’s protocol (ThermoFisher Scientific). For proliferation assay performed in the presence of siRNAs targeting RAGE, FAK and YAP, MDA-MB-231 and BT-549 TNBC cells overexpressing RAGE were transfected with each siRNA and then stimulated with rhS100A8/A9 (100ng/ml). After 72 hours, cell proliferation rate was evaluated by using Cell Proliferation Kit I (MTT) (#11465007001, MilliporeSigma) following the manufacturer instructions.

### Clonogenic assay

BT-549 TNBC cells overexpressing RAGE (1 × 10^3^) were seeded in 6-well plates and treated with rhS100A8/A9 (100ng/ml) alone or in combination with the RAGE antagonist FPS-ZM1 (1 μM) and FAK inhibitor VS-4718 (1 μM). For clonogenic assay performed in the presence of siRNAs, BT-549 TNBC cells overexpressing RAGE were transfected with siRNA targeting Control (20 nM) and siRNA targeting CTGF (20 nM), respectively, and after 72 hours cells were trypsinized, counted and 1 × 10^3^ cells were seeded in 6-well plates and treated with rhS100A8/A9 (100ng/ml), while remaining cells were processed to ascertain CTGF knockdown by RT-PCR. After 10 days, media from the wells was removed and cells were washed twice with 1X PBS, fixed for 5 minutes with methanol-acetic acid solution (3:1) and stained for 15 minutes with 0.5% crystal violet solution diluted in methanol. Lastly, wells were extensively washed with water and leave to dry at room temperature. Colony number was analyzed using Image-J program.

### Transwell migration assay

Migration assay was performed using boyden chambers (Costar Transwell® Permeable Supports, 5.0 μm polycarbonate membrane, 6.5 mm Insert, Corning Incorporated) in 24-well plates. Briefly, MDA-MB 231 and BT-549 TNBC cells overexpressing RAGE were seeded onto the upper membrane of the chamber at a density of 2.5 × 10^5^ cells/ml in serum-free medium. Next, the cells were stimulated with rhS100A8/A9 (100ng/ml) used alone or in combination with the RAGE antagonist FPS-ZM1 (1 μM) and FAK inhibitor VS-4718 (1 μM). For transwell migration assay performed in the presence of siRNAs, BT-549 TNBC cells overexpressing RAGE were transfected with siRNA targeting Control (20 nM) and siRNA targeting FLNA (20 nM), and after 72 hours cells were trypsinized, counted and 2.5 × 10^5^ cells were seeded onto the upper membrane of the chamber in serum-free medium and stimulated with rhS100A8/A9 (100ng/ml), while remaining cells were processed to ascertain FLNA knockdown by RT-PCR. 4 hours after incubation at 37 °C, medium was removed from the chambers, cells were washed twice with 1X PBS, fixed with 3.7% formaldehyde diluted in 1X PBS at room temperature for 2 minutes, washed twice with 1X PBS and permeabilized with 100% methanol at room temperature for 20 minutes. Then, methanol was removed, chambers were washed twice with 1X PBS and stained by using Giemsa solution for 15 minutes at room temperature. Finally, Giemsa solution was removed and chambers were washed twice with 1X PBS. No-migrated cells were removed by cotton swabs while migrated cells were counted under the microscope.

## Statistical analysis

The differences between experimental groups were analyzed using ANOVA and independent t-tests. The asterisks of figures denote statistical significance (* *p < 0.05*).

## Results

### S100A8/A9-RAGE system is remarkably expressed in TNBC and correlates with a poor clinical outcome in BC

**Fig. 1:**
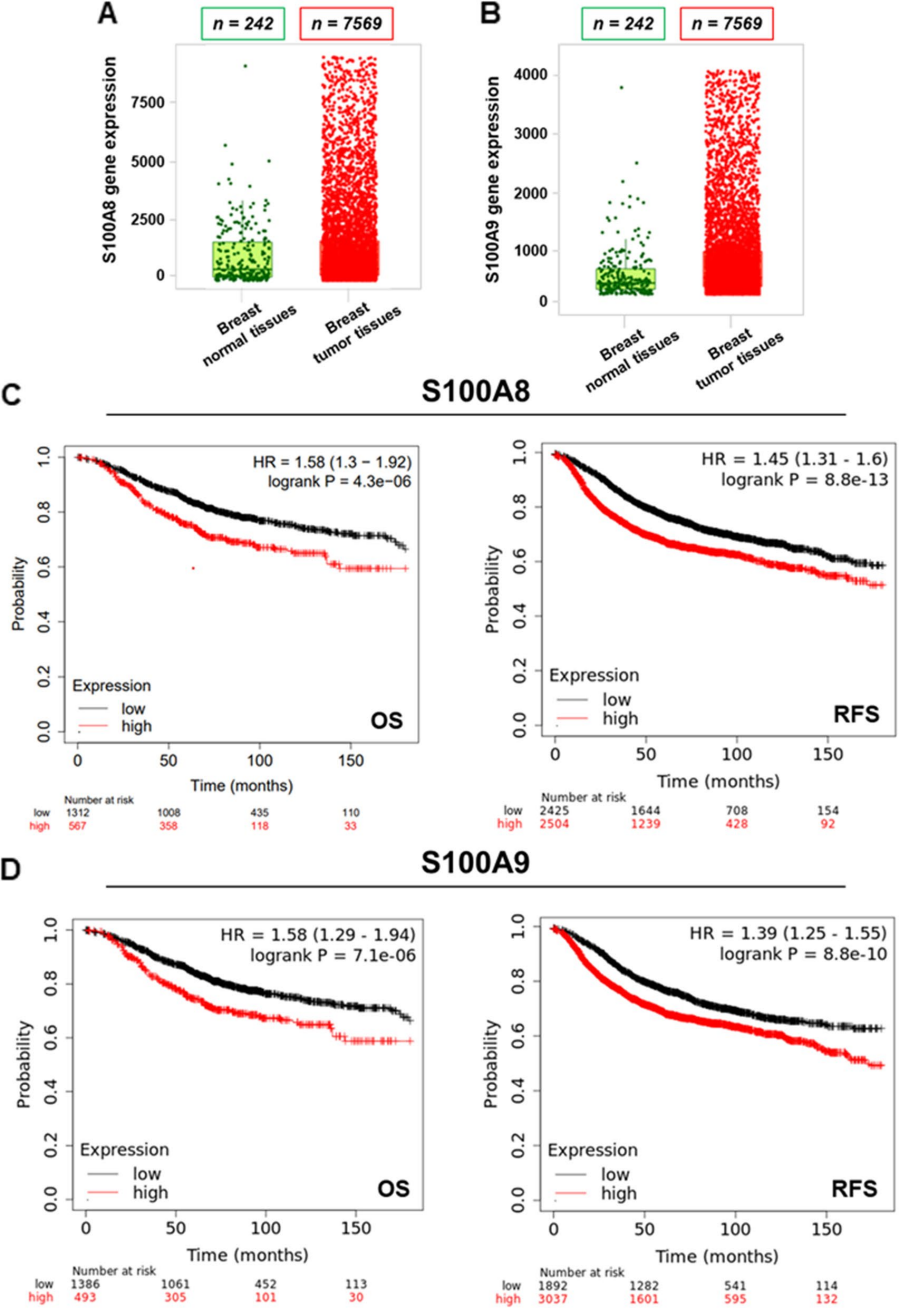
S100A8 and S100A9 are greatly expressed in primary breast tumors and correlate with poor clinical outcomes. **A** TNM box plot of S100A8 gene expression in normal and breast tumor tissues. **B** TNM box plot of S100A9 gene expression in normal and breast tumor tissues. **C** Kaplan-Meier Plotter Overall Survival (OS) (*n = 1879*) and Relapse Free Survival (RFS) (*n = 4929*) analysis in breast cancer subtypes harboring low and high levels of S100A8, respectively, at a follow up threshold of 180 months. Log-rank *p-value* is indicated within the boxes. All possible cutoff values between the lower and upper quartiles were automatically computed (i.e., auto select best cutoff on the website). Cutoff values used in the analysis were as follows: S100A8-OS: *3285*; S100A8-RFS: *639*. **D** Kaplan-Meier Plotter Overall Survival (OS) (*n = 1879*) and Relapse Free Survival (RFS) (*n = 4929*) in breast cancer subtypes harboring low and high levels of S100A9, respectively, at a follow up threshold of 180 months. Log-rank *p-value* is indicated within the boxes. All possible cutoff values between the lower and upper quartiles were automatically computed (i.e., auto select best cutoff on the website). Cutoff values used in the analysis were as follows: S100A9-OS: *2293*; S100A9-RFS: *278*

Dysregulated expression of Ca^2+^-dependent pro-inflammatory cytokines S100A8 and S100A9 has been associated with the onset of aggressive phenotypes in several tumors, including BC [41]. Likewise, the up-regulation of S100A8/A9 hetero-complex has been tightly linked to poor prognostic clinical indicators in BC patients [9, 42, 43]. Therefore, we began our study evaluating the expression levels and clinical significance of S1008 and S100A9 in BC by querying available online bioinformatics tools. Analyzing gene chip based data derived from TNM-plot analyzer (https://tnmplot.com/analysis/), we found significantly higher expression levels of both S100A8 and S100A9 in breast tumor samples respect to normal breast tissues (Fig. 1A-B). In addition, we detected a significant reduced expression of S100A8 and S100A9 when comparing BC metastatic samples with normal breast tissues (Additional file 1: Supplementary Fig. S1A-F). Moreover, the expression of S100A9 was found significantly down-regulated in BC metastatic samples respect to breast tumor samples (Additional file 1: Supplementary Fig. S1E-F). To gain a clinical perspective of the relevance of these findings, we first used the Kaplan-Meier Plotter analysis of Breast Cancer (https://kmplot.com/analysis/index.php?p=service&cancer=breast). This approach showed lower OS (*n = 1879)* as well as RFS (*n = 4929)* rates in BC patients harboring high levels of S100A8 and S100A9 with respect to patients exhibiting reduced levels (Fig. 1C-D). To strengthen this remarkable prognostic indication, we also conducted a meta-analysis study by elaborating clinical data derived from numerous BC datasets. This investigation nicely confirmed that high expression of both S100A8 and S100A9 is associated with a worse clinical outcome in BC patients (Additional file 2: Supplementary Fig. S2A-C). Aiming at further characterizing the significance of S100A8 and S100A9 in breast malignancy, we defined their expression pattern among the different breast tumor subclasses. Using the GEPIA2 database (http://gepia2.cancer-pku.cn/#index), we found higher expression levels of S100A8 and S100A9 in TNBC subtype compared to the normal breast counterpart (Additional file 3: Supplementary Fig. S3A-B). Moreover, we assessed a correlation between S100A8 and S100A9 expression levels in TNBC samples (Additional file 3: Supplementary Fig. S3C). On the contrary, Luminal-A and Luminal-B BC subtypes showed a lower expression of both S100A8 and S100A9 respect to normal breast tissues (Additional file 3: Supplementary Fig. S3D-E). Interestingly, comparing all BC subtypes we found that TNBC and HER2 positive BC subgroups exhibit the highest expression levels of S100A8 and S100A9, as determined exploring two diverse public BC datasets (Additional file 3: Supplementary Fig. S3F-G; Additional file 4: Supplementary Fig. S4A-C). Overall, these data suggest that S100A8 and S100A9 may be considered as prognostic indicators in HER2-positive and TNBC BC phenotypes.

**Fig. 2:**
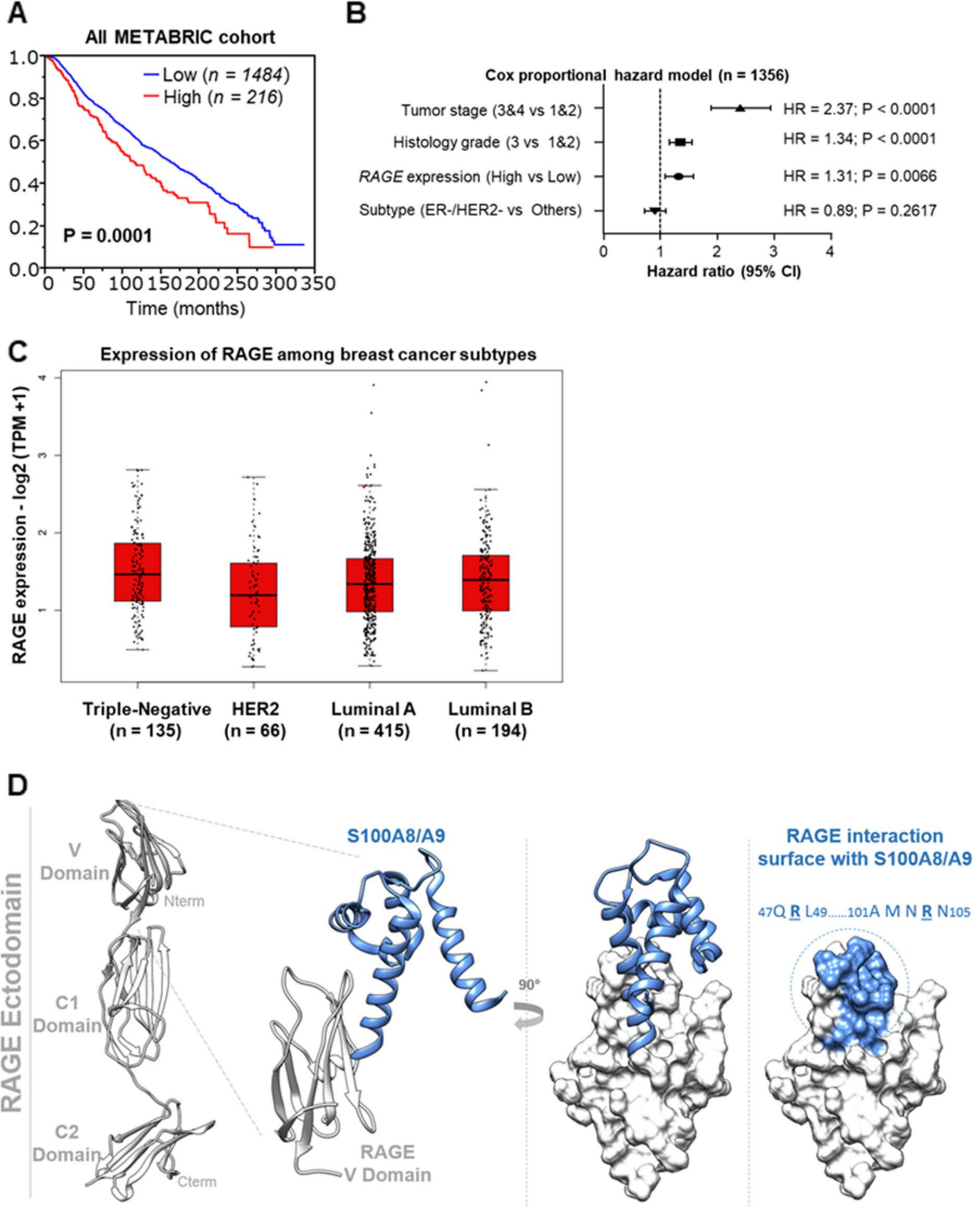
Increased RAGE expression correlates with worse prognostic hallmarks in breast cancer. **A** Median Overall Survival (OS) rate in breast cancer patients harboring high and low RAGE expression levels. *p-value* is shown within the box. **B** Multivariate Cox proportional hazard model linking RAGE expression pattern to breast tumor stage, histological grade and receptor expression pattern, respectively. **C** Gene Expression Profiling Interactive Analysis (GEPIA) box plot of RAGE in TNBC, HER2-positive, Luminal-A and Luminal-B breast cancer subclasses. **D** Working hypothesis postulating the potential putative binding site of S100A8/A9 on the V-domain of RAGE

RAGE has been proposed as a potential mediator of oncogenic effects elicited by S100A8/A9 complex in diverse malignancies, including BC [17, 44, 45]. Therefore, we focused on RAGE taking also into account its elevated expression levels in TNBCs and node-positive BC tissues and that RAGE expression increases with breast tumor size [46]. Evaluating the METABRIC Breast Cancer cohort (https://www.cbioportal.org/), we found that the OS in BC patients exhibiting high expression levels of RAGE was poorer respect to patients harboring low expression levels of RAGE (Fig. 2A). We also observed an association, although not significant, between high RAGE expression and reduced OS in BC through a meta-analysis network including different BC cohorts (Additional file 5: Supplementary Fig. S5A-B). Of clinical relevance, a multivariate cox proportional hazard model analysis revealed that RAGE expression was an independent prognostic factors in BC patients when we took three clinically important factor such as T-stage, histological grade and receptor expression pattern into consideration for the multivariate analysis (Fig. 2B). Then, exploring the GEPIA2 database (http://gepia2.cancer-pku.cn/#index), we observed higher (albeit not significant) expression levels of RAGE in TNBC respect to HER2-positive, Luminal-A and Luminal-B breast tumor subtypes (Fig. 2C). These findings suggest that RAGE may represent a candidate prognostic biomarker in breast malignancy. Altogether, our bioinformatics data prompted us to dissect the intracellular signaling network triggered by the S100A8/A9-RAGE system (Fig. 2D) in the context of the aggressive TNBC, for which there is an urgent need to identify novel signaling vulnerabilities toward more effective stratified therapies.

### S100A8/A9-RAGE system activates FAK through ROCK/myosin/Rho-A signaling in TNBC cells

**Fig. 3:**
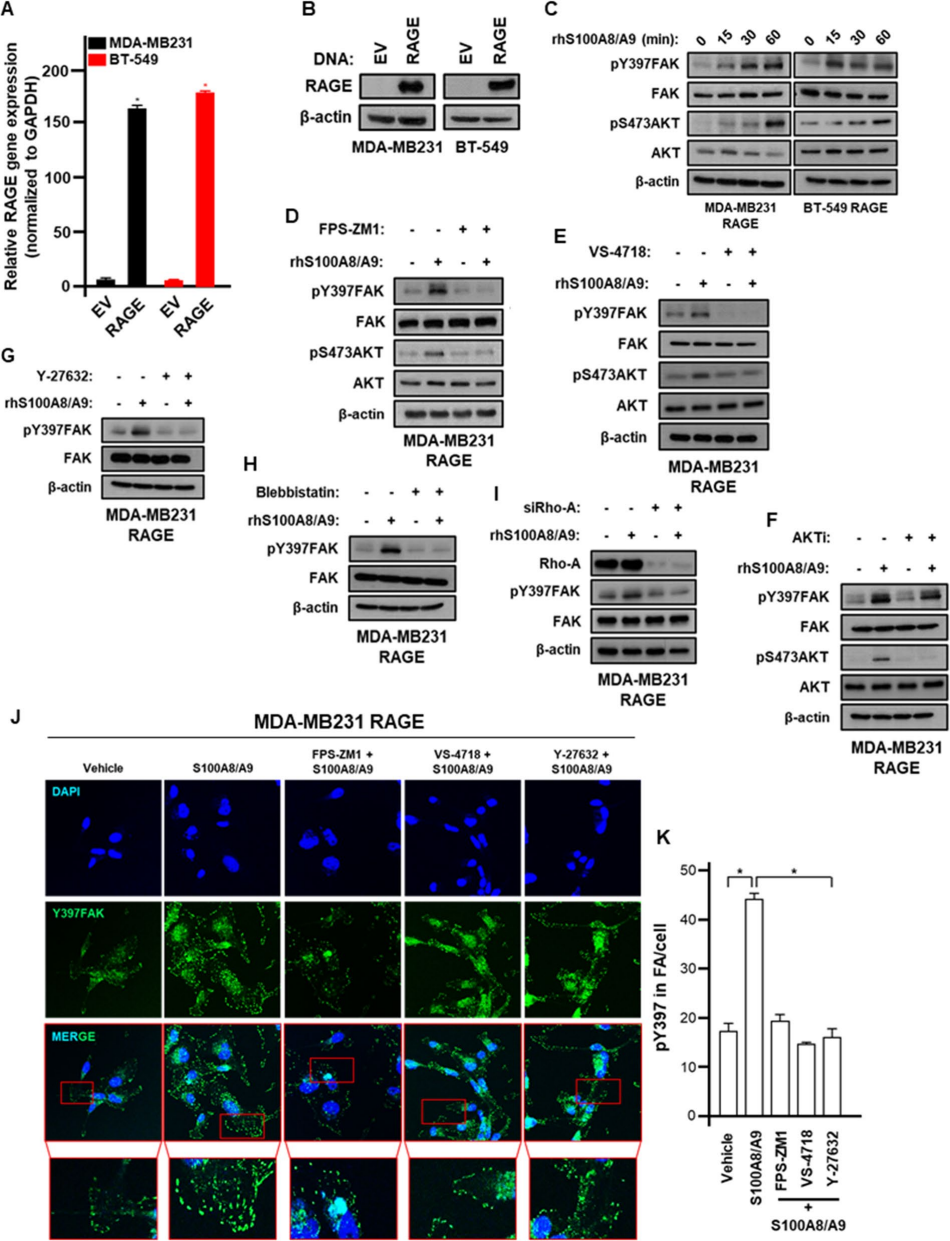
S100A8/A9-RAGE signaling activates FAK in TNBC cells. **A** RAGE mRNA levels in MDA-MB231 and BT-549 cells after infection with pCDNA3 and pcDNA3.RAGE DNA vectors. **B** RAGE immunoblot in MDA-MB231 and BT-549 cells after infection with pCDNA3 and pcDNA3.RAGE DNA vectors. **C** pY397FAK, FAK, pS473AKT and AKT immunoblots in MDA-MB231 and BT-549 cells overexpressing RAGE and treated with 100 ng/ml rhS100A8/A9 for the indicated times. **D** pY397FAK, FAK, pS473AKT and AKT immunoblots in MDA-MB231 cells overexpressing RAGE and treated for 30 minutes with 100 ng/ml rhS100A8/A9 alone or in combination with 1 μM RAGE antagonist FPS-ZM1. **E** pY397FAK, FAK, pS473AKT and AKT immunoblots in MDA-MB231 cells overexpressing RAGE and treated for 30 minutes with 100 ng/ml rhS100A8/A9 alone or in combination with 1 μM FAK inhibitor VS-4718. **F** pY397FAK, FAK, pS473AKT and AKT immunoblots in MDA-MB231 cells overexpressing RAGE and treated for 30 minutes with 100 ng/ml rhS100A8/A9 alone or in combination with 1 μM AKT inhibitor Ipatasertib. **G** pY397FAK and FAK immunoblots in MDA-MB231 cells overexpressing RAGE and treated for 30 minutes with 100 ng/ml rhS100A8/A9 alone or in combination with 1 μM ROCK inhibitor Y-27632. **H** pY397FAK and FAK immunoblots in MDA-MB231 cells overexpressing RAGE and treated for 30 minutes with 100 ng/ml rhS100A8/A9 alone or in combination with 1 μM Myosin II inhibitor Blebbistatin. **I** pY397FAK and FAK immunoblots in MDA-MB231 cells overexpressing RAGE and treated for 30 minutes with 100 ng/ml rhS100A8/A9 alone or in the presence of siRNA control and siRNA targeting Rho-A, respectively. **J** Y397FAK (green) and Nuclei (blue) confocal immunofluorescence staining performed in MDA-MB231 cells overexpressing RAGE and stimulated for 30 minutes with 100 ng/ml rhS100A8/A9 alone or in combination with 1 μM RAGE antagonist FPS-ZM1, 1 μM FAK inhibitor VS-4718 and 1 μM ROCK inhibitor Y-27632, respectively. **K** pY397 relative quantification in FA/cell. Error bars represent mean ± SD. * indicates *p-value* < 0.05. ꞵ-actin served as loading control for immunoblots. Results shown are representative of three independent experiments performed in triplicate

In order to explore the signaling network activated by S100A8/A9-RAGE system in TNBC, we established stable overexpressing RAGE MDA-MB 231 and BT-549 cells. The transduction of RAGE overexpression vector resulted in a robust upregulation of RAGE at both mRNA and protein levels (Fig. 3A-B). RAGE binding to S100A8/A9 was shown to promote chemoattractant-induced F-actin polymerization, thereby enhancing BC cell mesenchymal properties and EMT [17]. Among the pivotal gatekeepers involved in the regulation of breast tumor cell cytoskeleton and actin re-organization, FAK plays an essential role in mediating crucial signals from Focal Adhesions (FAs) to cell survival, migration and transcriptional programs [47–49]. In accordance with these findings, we observed the activation of FAK along with the phosphorylation of AKT upon S100A8/A9 treatment in TNBC cells overexpressing RAGE (Fig. 3C). These responses were prevented using the RAGE antagonist FPS-ZM1 (Fig. 3D) and the FAK inhibitor VS-4718 (Fig. 3E), whereas the AKT inhibitor ipatasertib was able to abolish only AKT phosphorylation (Fig. 3F), suggesting that S100A8/A9-RAGE system induces the activation of FAK that in turn triggers AKT phosphorylation in MDA-MB 231 TNBC cells. In order to establish a physiological condition, we interfered genetically with RAGE abrogating its expression. RAGE silencing abrogated FAK phosphorylation mediated by S100A/A9 in MDA-MB 231 and BT-549 TNBC cells (Additional file 6: Supplementary Fig. S6A-B). As observed in MDA-MB 231 cells, the pharmacological inhibition of RAGE and/or FAK prevented the activation of FAK triggered by S100A8/A9 also in BT-549 TNBC cells (Additional file 6: Supplementary Fig. S6C-D). Aiming to provide insights in the molecular mechanism involved in the activation of FAK by S100A8/A9-RAGE system, we performed further experiments using both pharmacological and genetic approaches in order to dissect mechanisms involved in the actin polymerization processes [50]. In this vein, both the ROCK inhibitor Y-27632 and the myosin-II inhibitor Blebbistatin were found to repress FAK activation triggered by S100A8/A9-RAGE system in TNBC cells (Fig. 3G-H, Additional file 6: Supplementary Fig. S6E). Likewise, Rho-A knockdown prevented the phosphorylation of FAK induced by S100A8/A9-RAGE axis (Fig. 3I). Aligned with these data, the number of FAs observed upon S100A8/A9-RAGE activation was reduced by RAGE, FAK and ROCK inhibition (Fig. 3J-K). Cumulatively, these findings suggest that an acto-myosin dependent pathway is involved in the activation of FAK mediated by S100A8/A9-RAGE system in TNBC cells (Additional file 6: Supplementary Fig. S6F).

### Hippo pathway is enriched in BC lesions harboring high RAGE expression levels

**Fig. 4:**
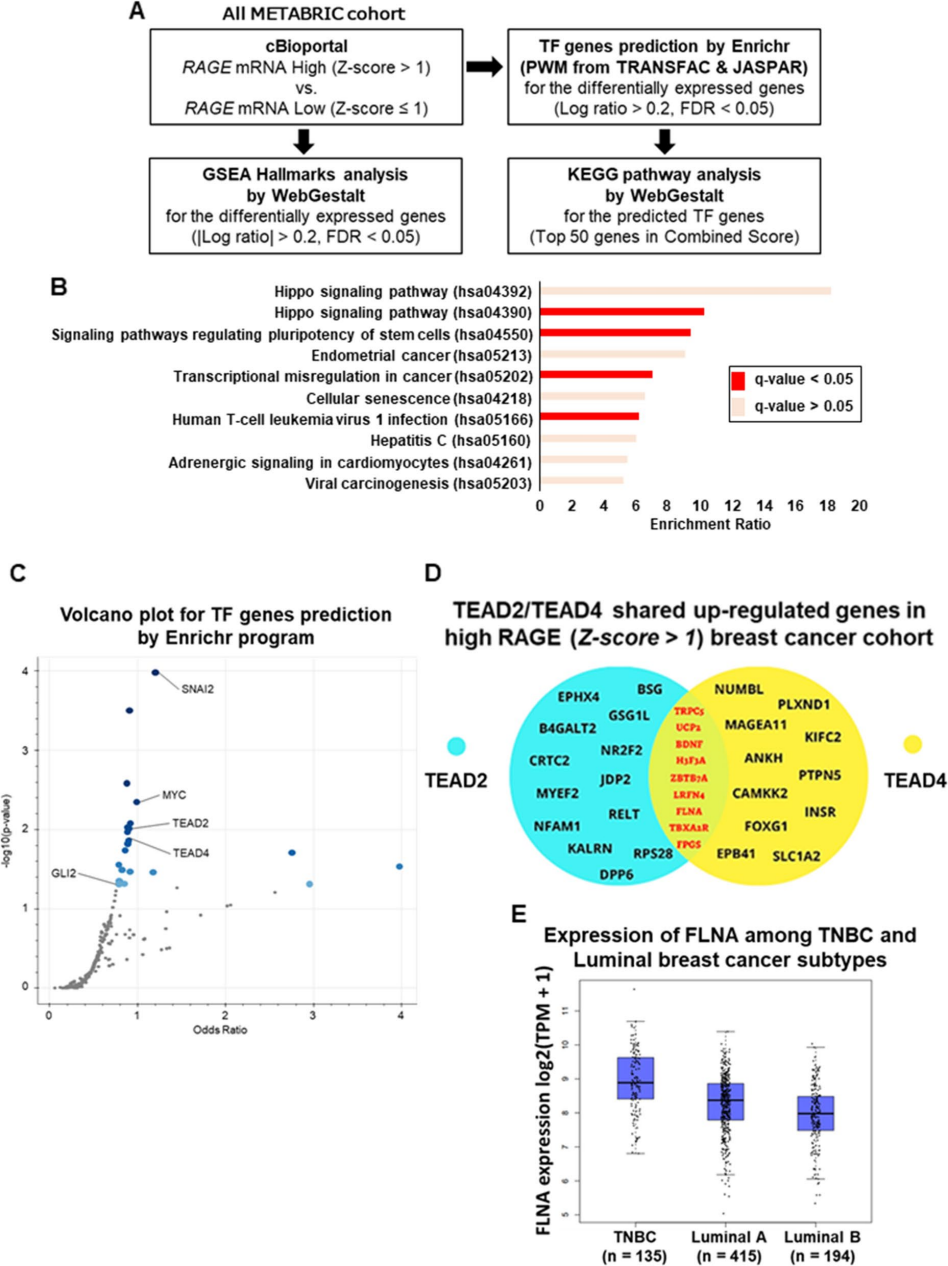
Hippo pathway is significantly enriched in high RAGE expression breast cancer cohort. **A** Differentially expressed genes (DEGs) between high (*Z-score > 1*) and low (*Z-score ≤ 1*) RAGE expression groups in breast cancer and estimation of GSEA Hallmark in DEGs using cBioportal and Webgestalt programs. **B** Putative significant pathways identified among the high RAGE (*Z-score > 1*) expression breast cancer group as indicated by the KEGG pathway analysis for the predicted TF genes based on the DEGs. **C** Volcano plot for TF genes prediction by Enrichr program in high RAGE (*Z-score > 1*) expression breast cancer cohort. The Volcano plot shows the significance of each potential TF gene predicted by Enrichr program based on the position weight matrices from JASPAR & TRANSFAC databases. The x-axis measures the odds ratio (0, inf) calculated for the TF genes, while the y-axis gives the -log10 (*p-value*) of the TF genes. Larger blue points represent significant TF genes (*p-value < 0.05*); smaller gray points represent non-significant TF genes. The darker blue color points means the highest significance. **D** Venn diagram of the shared up-regulated genes by TEAD2 and TEAD4 Hippo pathway TFs in high RAGE (*Z-score > 1*) expression breast cancer cohort. **E** Gene Expression Profiling Interactive Analysis (GEPIA) box plot of FLNA expression levels in TNBC, Luminal-A and Luminal-B breast cancer subtypes

We performed a bioinformatics analysis in order to identify molecular signatures potentially correlating with high RAGE expression levels in BC cohorts. In this vein, by exploring the METABRIC dataset in cBioportal (http://cbioportal.org) we first identified BC samples with high RAGE mRNA levels (*Z-score > 1*) respect to those exhibiting low RAGE expression levels (*Z-score ≤ 1*) (Fig. 4A) [29]. Using the WebGestalt program [32], we then filtered only differentially expressed genes (DEGs) between high and low RAGE expression BC subgroups (Fig. 4A). Subsequently, using the Enrichr analyzer [51], we identified the predicted Transcriptional Factors (TFs) whose function control the DEGs in the high RAGE expressing BC group, and used the WebGestalt program to identify pivotal KEGG pathways correlating with these TFs (Fig. 4A). Intriguingly, “Hippo signaling” was the most significant activated pathway in the high RAGE expression BC cohort (*q-value < 0.05*) (Fig. 4B). Precisely, “hsa04390” Hippo signaling pathway ID incorporates 157 genes respect to “hsa04392” Hippo signaling pathway ID, which combines 29 genes in the context of interspecies conservation (Fig. 4B). In both “hsa04390” and “hsa04392” Hippo signaling pathway IDs, the canonical Hippo pathway TFs TEAD2 and TEAD4 were found among the top 50 predicted TFs activated in the high RAGE expression BC cohort (Fig. 4C; Additional file 7: Supplementary Fig. S7A). In order to deepen our findings, we aimed to investigate the most relevant upregulated genes by TEAD2 and TEAD4 in the high RAGE expression BC cohort. To this end, we first established the target genes up-regulated by TEAD2 and TEAD4 (*Z-score > 1*), respectively (Additional file 7: Supplementary Fig. S7B), and then we filtered only the shared up-regulated genes by both TFs (*Z-score > 1*) (Fig. 4D). Among the identified up-regulated genes (Fig. 4D), we focused on the gene encoding the actin-crosslinking protein Filamin-A (FLNA), which plays a crucial role in both cytoskeleton remodeling and FAs turnover through the anchorage of a wide variety of adhesive proteins [52, 53]. FLNA has been closely associated with breast tumor progression and its inhibition has been shown to improve chemotherapy efficacy in TNBC [54, 55]. Considering that Filamin protein family consists of three different components (i.e. FLNA, FLNB and FLNC) [56], we sought to determine their expression pattern comparing TNBC and luminal BC subtypes. Higher FLNA expression was detected in TNBC respect to luminal BC subgroup (Fig. 4E), whereas FLNB as well as FLNC expression levels were found higher in luminal breast tumors respect to TNBC (Additional file 7: Supplementary Fig. S7C-D). In addition, analyzing data from diverse BC cohorts we found an association albeit not significant between high FLNA expression and reduced OS in BC patients (Additional file 8: Supplementary Fig. S8A-B). Collectively, our data suggest that Hippo signaling may be involved in S100A8/A9-RAGE transcription circuitry resulting in the up-regulation of several target genes, including FLNA.

### The S100A8/A9-RAGE system activates Hippo/YAP pathway in TNBC cells

**Fig. 5:**
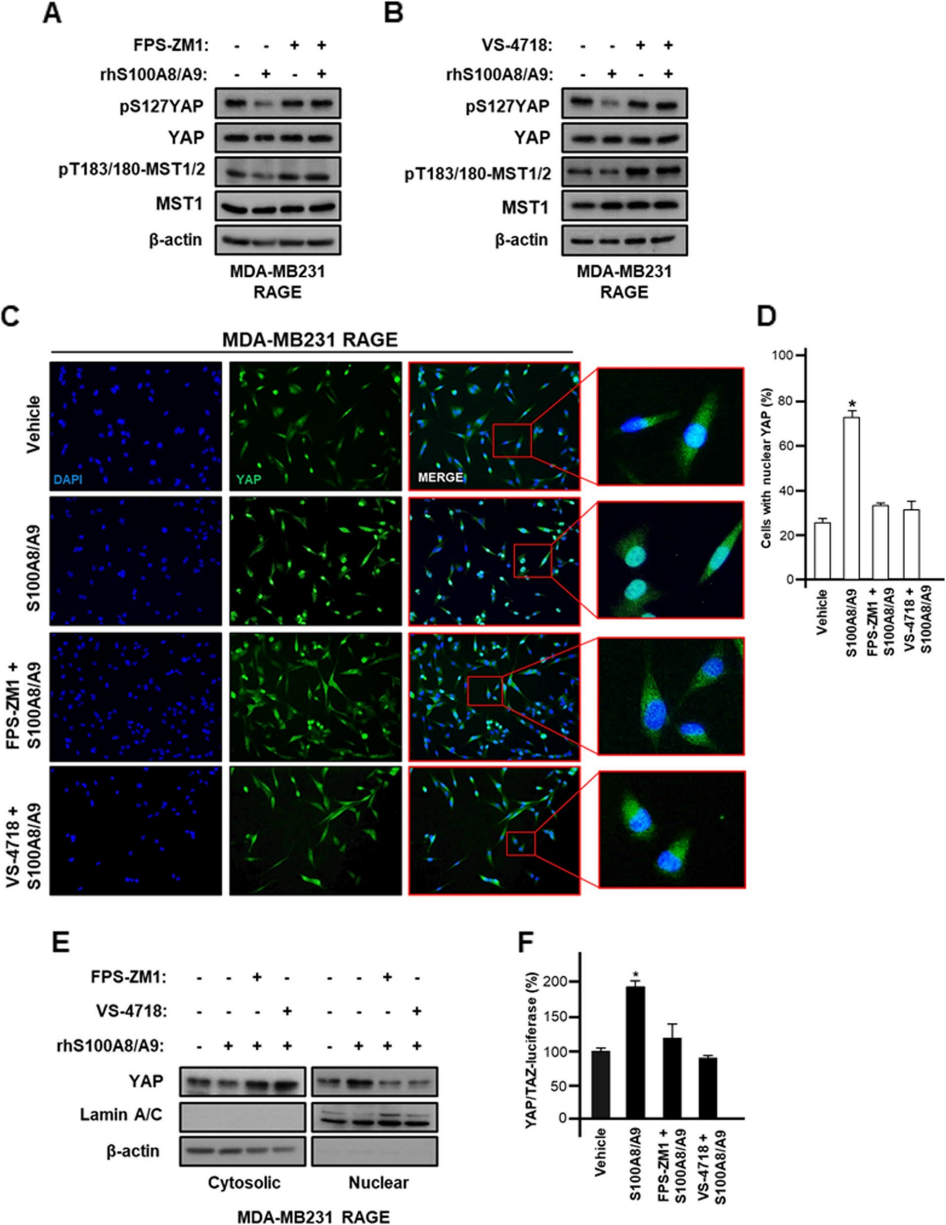
S100A8/A9-RAGE-FAK signaling triggers YAP activity in TNBC. **A** pS127YAP, YAP, pT183/180-MST1/2 and MST1 immunoblots in MDA-MB231 cells stable overexpressing RAGE and treated for 60 minutes with 100 ng/ml rhS100A8/A9 alone or in combination with 1 μM RAGE antagonist FPS-ZM1. **B** pS127YAP, YAP, pT183/180-MST1/2 and MST1 immunoblots in MDA-MB231 cells overexpressing RAGE and treated for 60 minutes with 100 ng/ml rhS100A8/A9 alone or in combination with 1 μM FAK inhibitor VS-4718. **C** YAP nuclear immunofluorescence staining in MDA-MB231 cells overexpressing RAGE and treated for 60 minutes with 100 ng/ml rhS100A8/A9 alone or in combination with 1 μM RAGE antagonist FPS-ZM1 or 1 μM FAK inhibitor VS-4718. **D** Relative quantification of cells with enhanced YAP nuclear localization. **E** YAP nuclear and cytoplasmic fractionation in MDA-MB231 cells overexpressing RAGE treated for 60 minutes with 100 ng/ml rhS100A8/A9 alone or in combination with 1 μM RAGE antagonist FPS-ZM1 or 1 μM FAK inhibitor VS-4718, using lamin A/C and ꞵ-actin as nuclear and cytoplasmic control markers, respectively. **F** YAP/TAZ luciferase reporter assay in MDA-MB231 cells overexpressing RAGE and treated for 6 hours with 100 ng/ml rhS100A8/A9 alone or in combination with 1 μM RAGE antagonist FPS-ZM1 or 1 μM FAK inhibitor VS-4718. Error bars represent mean ± SD. * indicates *p-value* < 0.05. ꞵ-actin served as loading control for immunoblots. Results shown are representative of three independent experiments performed in triplicate

YAP is a key mediator of the Hippo pathway driving oncogenic effects in TNBC [23, 57]. The mechanisms triggering YAP activation in TNBC remain to be fully understood, albeit previous studies have identified certain YAP regulators [58, 59]. For instance, FAK has recently emerged as an important gatekeeper regulating YAP nuclear translocation and gene transcriptional programs toward uncontrolled growth effects and metastatic processes [20, 49]. Therefore, we attempted to examine whether S100A8/A9-RAGE system along with FAK can activate YAP in TNBC cells overexpressing RAGE. Notably, we found that S100A8/A9 exposure reduces YAP phosphorylation, thereby reflecting its activation in TNBC cells (Fig. 5A-B; Additional file 9: Supplementary Fig. S9A-B) [60]. Moreover, S100A8/A9-mediated YAP activation was reversed by inhibiting either RAGE (Fig. 5A; Additional file 9: Supplementary Fig. S9A) or FAK (Fig. 5B; Additional file 9: Supplementary Fig. S9B). To confirm the activation of YAP signaling in our model system, we assessed that S100A8/A9 treatment abrogates the phosphorylation of MST1/2, which may act as main core regulatory proteins in Hippo pathway (Fig. 5A-B). Of note, this effect was no longer evident by interfering with RAGE (Fig. 5A) or FAK (Fig. 5B). Next, we performed knock-down experiments to further assess the involvement of S100A8/A9/RAGE/FAK axis in the control of YAP activation. In this vein, we determined that the silencing of RAGE as well as FAK expression prevents YAP activation induced by S100A8/A9 in TNBC cells over-expressing RAGE (Additional file 9: Supplementary Fig. S9C-D). Likewise, the nuclear accumulation of YAP induced by S100A8/A9 was blunted interfering with RAGE and FAK, as determined through the quantification of YAP immunofluorescence staining (Fig. 5C-D) and analyzing nuclear/cytoplasmic cellular fraction (Fig. 5E). Under-phosphorylated and activated YAP shifts from cytoplasm into nucleus, where it binds to TEAD transcription factors acting as a co-activator for gene expression responses [61]. Therefore, the functional impact of S100A8/A9-RAGE-FAK system on YAP was confirmed by YAP/TAZ luciferase reporter assays (Fig. 5F). In addition, the S100A8/A9-regulated expression of Hippo/YAP canonical targets, CTGF and Cyr61, was abrogated targeting RAGE and FAK (Additional file 10: Supplementary Fig. S10A-D).

**Fig. 6:**
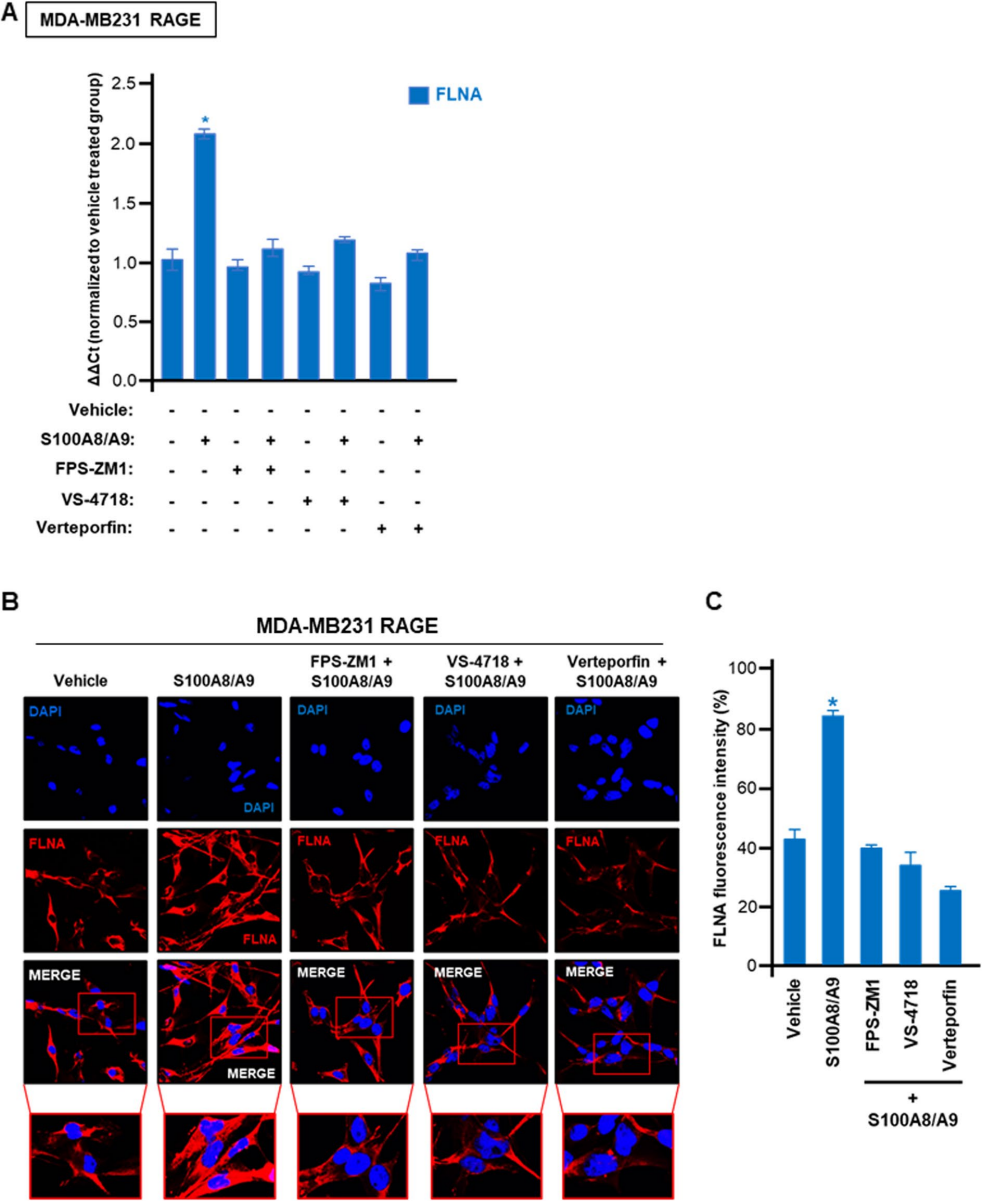
FLNA is upregulated by S100A8/A9-RAGE-FAK-YAP transduction network in TNBC. **A** FLNA mRNA levels in MDA-MB231 cells overexpressing RAGE and treated for 6 hours with 100 ng/ml rhS100A8/A9 alone or in combination with 1 μM RAGE antagonist FPS-ZM1, 1 μM FAK inhibitor VS-4718 and 1 μM YAP/TEAD disruptor Verteporfin. **B** FLNA (red) and Nuclei (blue) confocal immunofluorescence staining in MDA-MB231 cells overexpressing RAGE and stimulated for 6 hours with 100 ng/ml rhS100A8/A9 alone or in combination with 1 μM RAGE antagonist FPS-ZM1, 1 μM FAK inhibitor VS-4718 and 1 μM YAP/TEAD disruptor Verteporfin, respectively. **C** Relative percentage of FLNA fluorescence intensity. Error bars represent mean ± SD. * indicates *p-value* < 0.05. Results shown are representative of three independent experiments performed in triplicate

Considering that FLNA was identified among the top up-regulated genes by TEAD2/TEAD4 Hippo pathway transcription factors in the high RAGE expression BC cohort (See Fig. 4), we sought to assess whether S100A8/A9-RAGE-FAK signaling along with YAP can regulate the expression of FLNA in TNBC cells. Of note, the S100A8/A9-induced mRNA expression of FLNA was prevented using the RAGE antagonist FPS-ZM1 or the FAK inhibitor VS-4718 as well as disrupting YAP-TEAD interaction by using verteporfin (Fig. 6A). Corroborating these results, we also analyzed the expression of FLNA by immunofluorescence microscopy. The increased abundance as well as the median fluorescence intensity of cytoplasmic FLNA observed upon S100A8/A9 exposure, were significantly reduced by the pharmacological inhibition of RAGE, FAK and YAP-TEAD binding (Fig. 6B-C). Taken together, these results suggest that RAGE and FAK regulate YAP activity in TNBC cells, resulting in increased FLNA expression among other Hippo pathway-target genes.

### S100A8/A9-RAGE-FAK-YAP transduction signaling stimulates growth and migration of TNBC cells

**Fig. 7:**
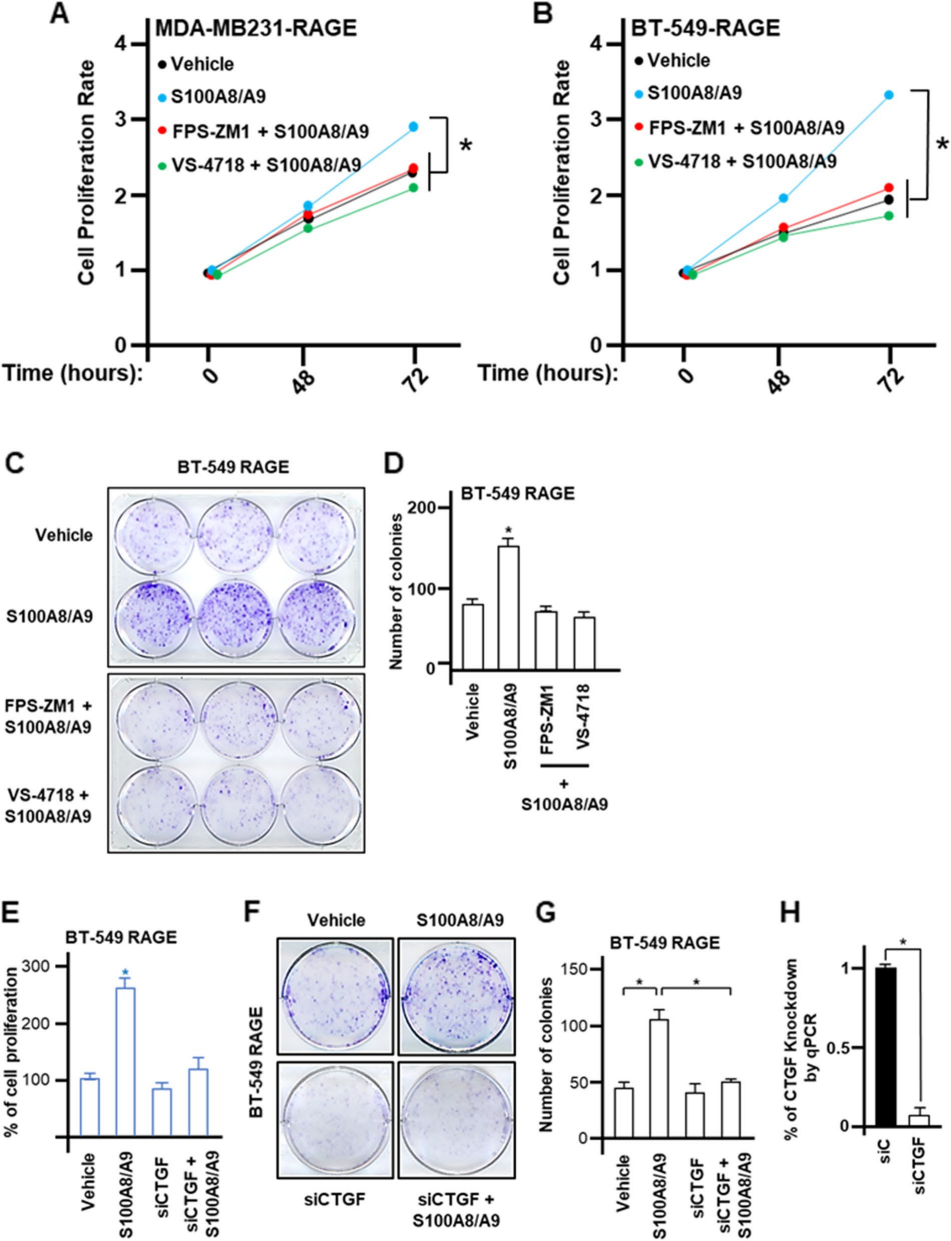
S100A8/A9-RAGE system prompts growth effects in TNBC cells. **A** Cell proliferation in MDA-MB231 cells overexpressing RAGE upon stimulation for 72 hours with 100 ng/ml rhS100A8/A9 used alone or in combination with 1 μM RAGE antagonist FPS-ZM1 or 1 μM FAK inhibitor VS-4718. **B** Cell proliferation in BT-549 cells overexpressing RAGE upon stimulation for 72 hours with 100 ng/ml rhS100A8/A9 used alone or in combination with RAGE antagonist 1 μM FPS-ZM1 or 1 μM FAK inhibitor VS-4718. **C**-**D** Colony formation assay in BT-549 cells overexpressing RAGE and treated for 10 days with 100 ng/ml rhS100A8/A9 alone or in combination with 1 μM RAGE antagonist FPS-ZM1m or 1 μM FAK inhibitor VS-4718. **E** Cell proliferation in BT-549 cells overexpressing RAGE and transfected with siRNA control (20 nM) and siRNA targeting CTGF (20 nM) and stimulated for 72 hours with 100 ng/ml rhS100A8/A9. **F**-**G** Colony formation assay in BT-549 cells overexpressing RAGE transfected with siRNA control (20 nM) and siRNA targeting CTGF (20 nM) and stimulated for 10 days with 100 ng/ml rhS100A8/A9. **H** CTGF knockdown efficiency is shown. Error bars represent mean ± SD. * *p-value* < 0.05 indicates. Results shown are representative of three independent experiments performed in triplicate

The S100A8/A9 complex promotes oncogenic transcriptional activities and contributes to BC metastasis [62, 63]. On the other hand, the growth of BC cells as well as TNBC tumor progression has been halted targeting RAGE by different approaches such as using monoclonal antibody therapy, and genetic and/or pharmacological strategies [15, 16]. FAK inhibition also suppressed breast primary tumor development and anchorage-independent growth of TNBC cells [64, 65]. Consistent with these observations, in the present study we ascertained that upon S100A8/A9 stimulation the proliferative responses of TNBC cells over-expressing RAGE were prevented by RAGE or FAK pharmacological inhibition (Fig. 7A-B) as well as knocking down the expression of RAGE, FAK and YAP (Additional file 11: Supplementary Fig. S11A-D). Likewise, targeting RAGE and FAK lowered the number of colonies triggered by S100A8/A9 treatment (Fig. 7C-D). In addition, the growth stimulation induced by the S100A8/A9-RAGE system was blunted silencing the expression of the Hippo/YAP canonical target CTGF (Fig. 7E-H). Together, these data suggest that S100A8/A9-RAGE system engages the downstream FAK-Hippo/YAP signaling to promote the growth of TNBC cells.

**Fig. 8:**
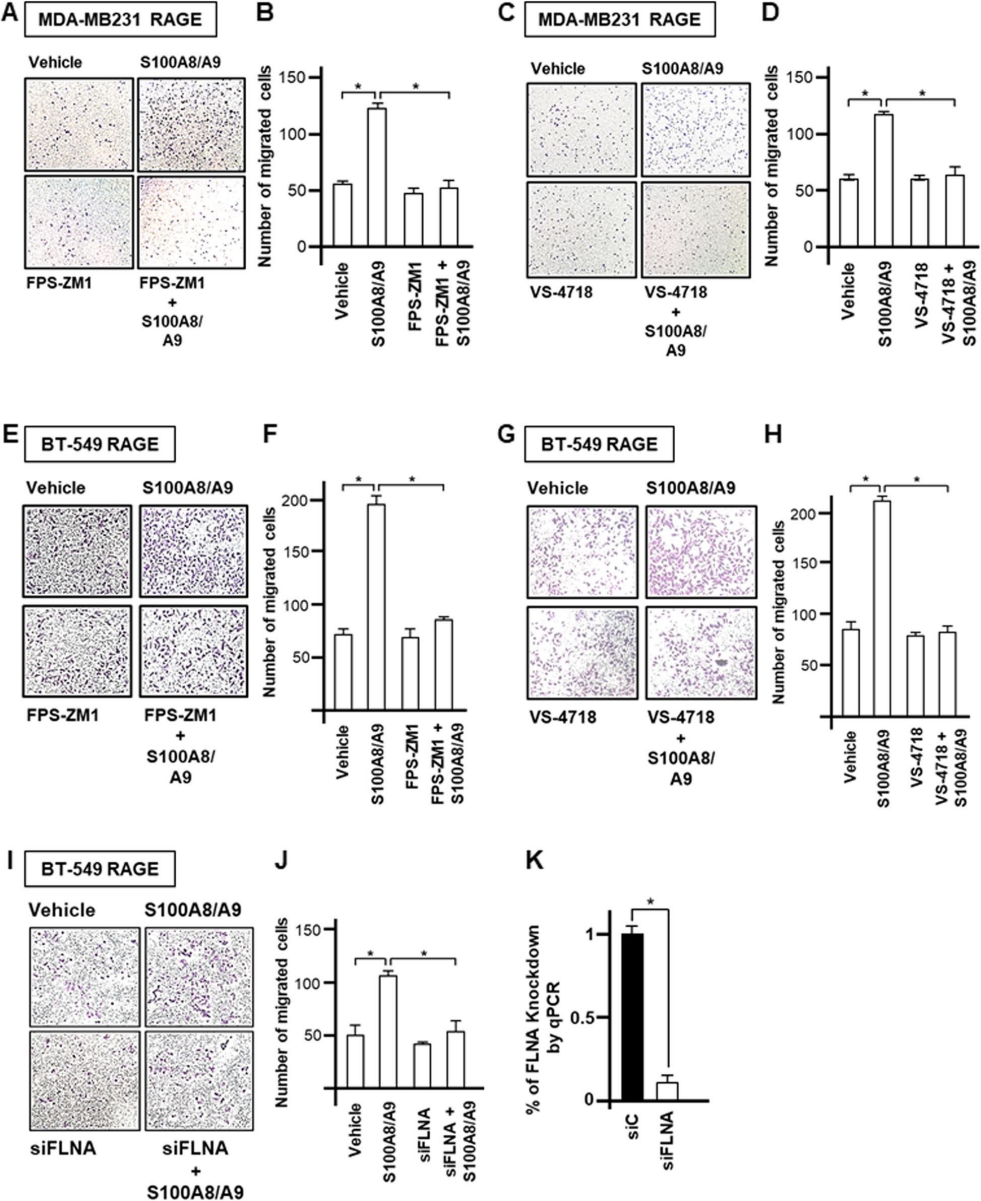
S100A8/A9-RAGE system increases the migration of TNBC cells. **A**-**B** Representative images (left) and relative quantification (right) of transwell migration in MDA-MB231 cells overexpressing RAGE and treated for 4 hours with 100 ng/ml rhS100A8/A9 alone or in combination with 1 μM RAGE antagonist FPS-ZM1. **C**-**D** Representative images (left) and relative quantification (right) of transwell migration in MDA-MB231 cells overexpressing RAGE and treated for 4 hours with 100 ng/ml rhS100A8/A9 alone or in combination with 1 μM FAK inhibitor VS-4718. **E**-**F** Representative images (left) and relative quantification (right) of transwell migration in BT-549 cells overexpressing RAGE and treated for 4 hours with 100 ng/ml rhS100A8/A9 alone or in combination with 1 μM RAGE antagonist FPS-ZM1. **G**-**H** Representative images (left) and relative quantification (right) of transwell migration in BT-549 cells overexpressing RAGE and treated for 4 hours with 100 ng/ml rhS100A8/A9 alone or in combination with 1 μM FAK inhibitor VS-4718. **I**-**J** Representative images (left) and relative quantification (right) of transwell migration in BT-549 cells overexpressing RAGE transfected with siRNA control (20 nM) and siRNA targeting FLNA (20 nM) and stimulated for 4 hours with 100 ng/ml rhS100A8/A9. **K** FLNA knockdown efficiency is shown. Error bars represent mean ± SD. * indicates *p-value* < 0.05. Results shown are representative of three independent experiments performed in triplicate

**Fig. 9:**
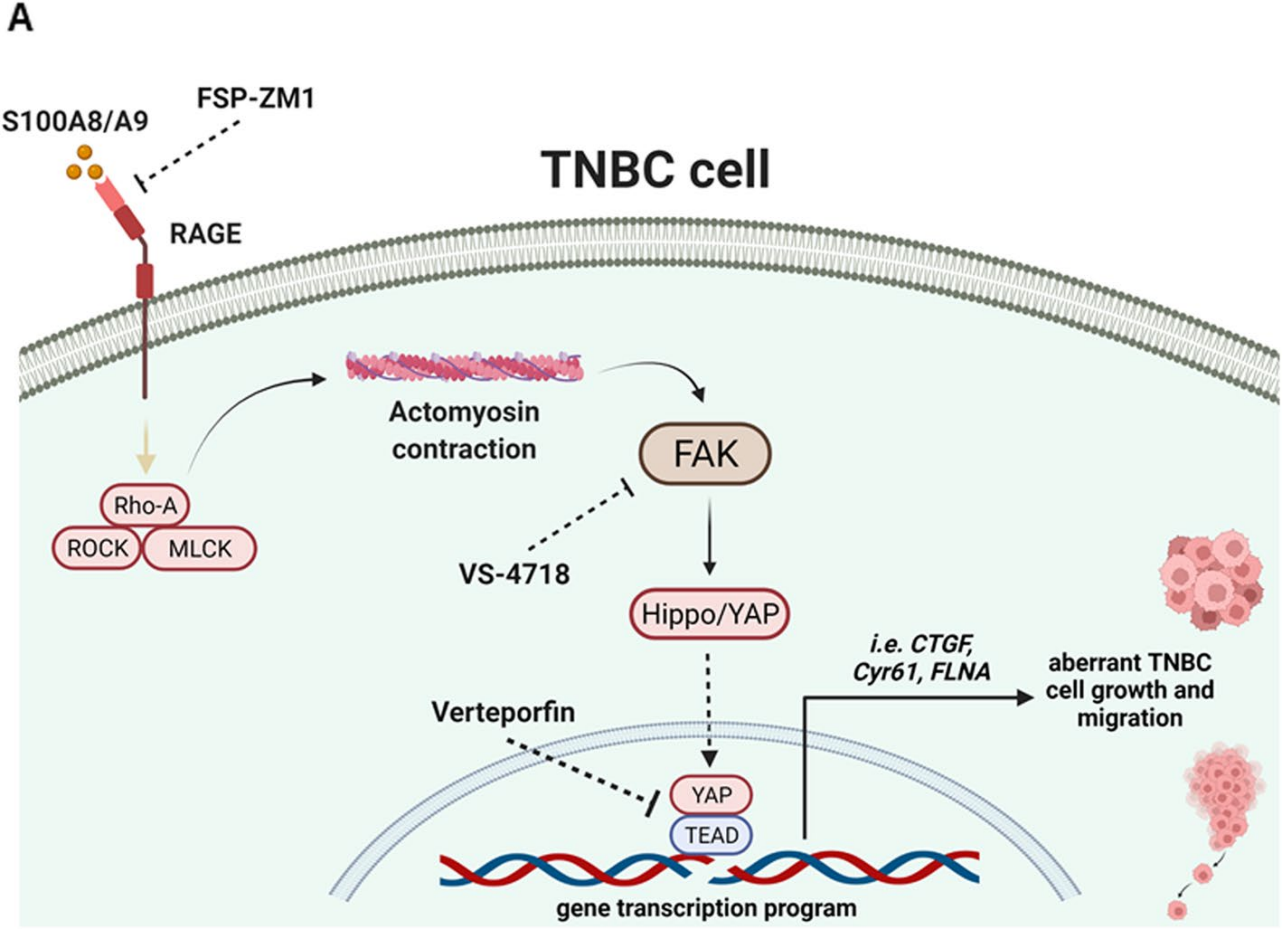
S100A8/A9-RAGE-FAK-YAP signaling in TNBC cells. **A** Cartoon depicting the proposed S100A8/A9-RAGE-FAK-YAP transduction network in TNBC cells. Targeting RAGE along with FAK/YAP-dependent transcriptional programs may disable the growth and migration of TNBC cells

Considering that the interaction between S100 Ca^2+^-binding proteins and RAGE promotes tumor progression also stimulating invasive effects [44, 66–69], we next aimed to explore whether S100A8/A9-RAGE activation prompts the migration of TNBC cells. S100A8/A9 treatment stimulated migratory effects in TNBC cells overexpressing RAGE, however these responses were reduced targeting either RAGE or FAK and by knocking-down the expression of the YAP/TEAD target FLNA (Fig. 8A-K). Collectively, our findings suggest that S100A8/A9-RAGE system induces aberrant growth and migration of TNBC cells by engaging both FAK and Hippo/YAP signal transduction mediators (Fig. 9A).

## Discussion

The development and widespread advances on ‘omics’ technologies are consistently improving the characterization of the clinical and molecular heterogeneity of TNBC ecosystem, which remains the BC subtype with a poorer differentiation, and the highest early tendency of metastasis and recurrence than other BC phenotypes [70–72]. Indeed, metastatic spread is the main hurdle for the therapeutic benefits in patients with early and advanced TNBC [73]. The combination of ICIs with cytotoxic chemotherapeutic agents provides new therapeutic opportunities for TNBC patients [74, 75]. However, there is still a compelling need to identify novel prognostic biomarkers that be also useful as potential molecular targets that might synergize with immunotherapy and improve personalized therapeutic responses in TNBC patients. For instance, an aberrant expression of S100A8 and S100A9 Ca^2 + −^-binding proteins has been detected in BC cohorts and correlated with the loss of ER in breast malignancy [9, 76]. Mechanistically, S100A8/A9 hetero-complex can drive oncogenic signaling pathways and transcriptional responses toward the onset of breast malignant phenotypes [62, 63]. However, the elucidation of S100A8/A9-mediated downstream transduction mechanisms, particularly in the high metastatic TNBC subtype, has not been yet fully explored.

Here, we first showed that the up-regulation of S100A8 and S100A9 expression is significantly associated with worse survival rates in BC patients and highly expressed in HER2-positive and TNBC rather than Luminal BC subtypes. We then assessed that the over-expression of RAGE, which acts as one of the main binding surface receptor for S100A8/A9 heterodimer [77], correlates with a poor OS in BC patients and is also prominently detected in TNBC. Taking advantage of TNBC cells engineered to stably overexpress RAGE, we have unveiled novel insights on the molecular mechanisms by which S100A8/A9-RAGE system may act in TNBC. In this regard, we found that S100A8/A9-induced RAGE activation primes FAK phosphorylation as well as the formation of FAs that in turn promote YAP nuclear accumulation and gene transcription programs. Biologically, we found that the S100A8/A9-RAGE-FAK-YAP signaling circuitry promotes the growth and migratory responses of TNBC cells.

Evidence from pre-clinical and clinical investigation have established S100A8 and its cognate-binding partner S100A9 as a potential prognostic biomarkers for reactivation of dormant tumor cells, prediction of metastatic risk and therapeutic responses failure in several malignancies, including BC [78–81]. Aligned with these studies, a poor OS in BC patients has been observed in the presence of high S100A9 expression levels [76], whereas higher S100A8 expression in both stromal and BC cells has been associated with worse clinical outcomes [82]. Of note, our bioinformatics investigation has revealed that S100A8 and S100A9 are greatly expressed in breast tumor tissues rather than in its normal counterpart. This clinical feature was not evident analyzing the expression of both S100A8 and S100A9 in BC metastatic tissues, probably due to the heterogeneity of expression patterns as well as the low number of metastatic samples. Remarkably, we found that high expression of both S100A8 and S100A9 correlates with a decreased OS and RFS rates in BC patients. Moreover, through the in depth analysis of large publicly available databases, we show that TNBC, unlike Luminal-A and Luminal-B BC subtypes, harbors higher levels of both S100A8 and S100A9 respect to non-tumor breast tissues. In addition, we found increased levels of S100A8 and S100A9 in TNBC and HER2-positive BC subtypes compared to Luminal-A and Luminal-B breast tumor subgroups, respectively, and we also highlight a strong correlation between S100A8 and S100A9 gene expression levels in TNBC.

Several pre-clinical studies have indicated RAGE as an attractive pharmacological target to halt primary tumor growth and recurrence of metastatic lesions [83–85]. Although not much has been determined about the predictive clinical significance and functional role of RAGE in BC, previous findings have proposed RAGE as a potential candidate biomarker for breast tumor development and progression, and as a promising therapeutic approach for the high invasive TNBC [14–16]. In the present study, we show that high RAGE expression levels are significantly correlated with worse survival in BC patients. We also noticed that increased RAGE expression may serve as an additional covariate to be included among the prognostic factors in BC patients. Further investigating the expression of RAGE among the BC subtypes, we showed an enhanced, albeit not significant, expression of RAGE in TNBC, thus confirming previous studies detecting high RAGE levels in TNBC tissues [15].

RAGE is a multi-ligand pattern recognition receptor binding to several ligands such as advanced glycation end products (AGEs), high mobility group box-1 peptide (HMGB-1), amyloid-β peptides and the S100-Ca^2+^ family proteins [86, 87]. Upon stimulation, RAGE activates several oncogenic signaling pathways and gene expression programs which in turn promote pro-inflammatory responses and malignant progression [88–90]. Particularly, interfering with RAGE-ligand mediated signaling impairs cell viability, adhesion, migration and invasion of TNBC cells [17, 91–93]. Here, we have elucidated a novel intracellular molecular signaling network triggered by S100A8/A9-RAGE system in TNBC by using TNBC cell lines stably engineered to overexpress RAGE. We demonstrate that S100A8/A9-RAGE system triggers FAK as well as AKT activation in TNBC cells, as evaluated using selective pharmacological agents and genetic approaches. In order to further dissect the mechanism by which S100A8/A9-RAGE system primes FAK activation, we determined the engagement of ROCK/myosin/Rho-A cytoskeleton mechanical transducers, strengthening previous studies showing the involvement of cytoskeletal partners in the mechanical stretch-dependent FAK phosphorylation [94, 95].

Although FAK has emerged as a key upstream mediator in controlling the mechanical cue-driven Hippo/YAP pathway activities in diverse malignant frameworks [49, 96, 97], the role of S100 Ca^2+^-proteins/RAGE system in this context has not been yet fully investigated. Only recently, it has been demonstrated that AGEs-RAGE may cooperate with the integrin receptors in promoting YAP nuclear accumulation through an enriched glycated extracellular matrix-dependent manner [25]. Intriguingly, in our study we have discovered that Hippo pathway is the most enriched signaling in BC patients with high RAGE expression respect to those patients with low levels of RAGE. Moreover, we have also identified the main significant up-regulated Hippo-TFs in BC patients harboring high RAGE expression levels and, among the downstream target genes up-regulated by the canonical YAP interactors TEAD2 and TEAD4 Hippo TF family members, we have detected FLNA as the pivotal downstream gene correlated to extracellular matrix or cytoskeleton-related pathways leading to cancer cell invasive features. Nicely fittings with these findings, we found that FLNA, unlike FLNB and FLNC, is highly expressed in TNBC rather than in Luminal BC subtypes. Furthermore, we determined that the S100A8/A9-RAGE system along with FAK trigger YAP activation and subsequent nuclear accumulation, thereby turning-on the transcription of Hippo canonical targets CTGF and Cyr61, as well as FLNA. Overall, our study supports the existence of a novel S100A8/A9-RAGE-FAK signaling mechanism controlling the Hippo/YAP pathway and its downstream targets, thereby increasing the growth and migration of TNBC cells. Recently, the discovery of a novel molecular framework has revealed that S100-Ca^2+^ binding proteins along with RAGE may contribute to the development of radiotherapy-resistant brain metastatic cancer cells, which were derived from different primary tumors including TNBC [98]. Interestingly, a significant reduction of metastasis in a TNBC brain metastatic syngeneic mouse model was observed lowering S100A9 levels along with whole-brain radiotherapy (WBRT) treatment [98]. Targeting the S100A9-RAGE axis with the antagonist FPS-ZM1 resulted in potentiated benefits of radiation without evidence of increased toxicity, therefore providing the rationale for a novel combination therapy to overcome radio-resistance in brain metastasis [98]. In accordance with these findings, our data raise the possibility to target the S100A8/A9-RAGE-FAK-Hippo/YAP axis for novel therapeutic intervention in TNBC patients, as depicted in Fig. 9**.**

## Conclusion

Our study has revealed that both S100A8/A9 and RAGE correlate with poor clinical outcomes in BC patients and are highly expressed in TNBC subtype. Mechanistically, S100A8/A9-RAGE system promotes FAK phosphorylation and regulates the Hippo pathway, thus increasing YAP nuclear localization and gene transcription activities that stimulate the growth and migration of TNBC cells. Therefore, the comprehensive analysis of S100-Ca^2+^ binding proteins and RAGE expression profile could be useful for the assessment of BC diagnosis, prognosis and new therapeutic strategies halting TNBC progression.

## Supplementary Information

**Additional file 1: Supplementary Fig. S1.** Expression of S100A8 and S100A9 in metastatic BC. (A) TNM box plot of S100A8 gene expression in normal (*n = 242*) and metastatic (*n = 82*) breast tissues. (B) TNM box plot of S100A8 gene expression in tumor (*n = 7569*) and metastatic (*n = 82*) breast tissues. (C) S100A8 expression levels in normal vs tumor, tumor vs metastatic, normal vs metastatic breast tissues, as evaluated by the Mann-Whitney U test. *p-value* is indicated within the box. (D) TNM box plot of S100A9 gene expression in normal (*n = 242*) and metastatic (*n = 82*) breast tissues. (E) TNM box plot of S100A9 gene expression in tumor (*n = 7569*) and metastatic (*n = 82*) breast tissues. (F) S100A9 expression levels in normal vs tumor, tumor vs metastatic, normal vs metastatic breast tissues, as evaluated by the Mann-Whitney U test. *p-value* is indicated within the box.

**Additional file 2: Supplementary Fig. S2.** Expression levels of S100A8 and S100A9 correlate with a worse overall survival tendency in BC patients. (A) Evaluation of overall survival in BC tumors exhibiting high S100A8 expression, as evaluated by a meta-analysis including 18 BC datasets. (B) Evaluation of overall survival in BC tumors exhibiting high S100A9 expression, as evaluated by a meta-analysis including 18 BC datasets. (C) Random effect model in BC tumors exhibiting high S100A8 and S100A9 expression levels. The value of each parameter is indicated within the box.

**Additional file 3: Supplementary Fig. S3.** S100A8 and S100A9 expression levels in the different subtypes of BC. (A) Gene Expression Profiling Interactive Analysis (GEPIA) box plot of S100A8 expression in TNBC samples respect to normal breast samples. * indicates *p*-value Cutoff of 0.01. (B) Gene Expression Profiling Interactive Analysis (GEPIA) box plot of S100A9 expression in TNBC samples respect to normal breast samples. * indicates *p*-value Cutoff of 0.01. (C) Correlation between S100A8 and S100A9 expression levels in TNBC. (D) Gene Expression Profiling Interactive Analysis (GEPIA) box plots of S100A8 expression in Luminal-A samples respect to normal breast samples, and Luminal-B samples respect to normal breast samples. * indicates *p*-value Cutoff of 0.01. (E) Gene Expression Profiling Interactive Analysis (GEPIA) box plots of S100A9 expression in Luminal-A samples respect to normal breast samples, and Luminal-B samples respect to normal breast samples. * indicates *p*-value Cutoff of 0.01. (F) Gene Expression Profiling Interactive Analysis (GEPIA) box plots of S100A8 expression in TNBC, HER2 positive, Luminal-A and Luminal-B breast tumor subtypes. * indicates *p*-value Cutoff of 0.01. (G) Gene Expression Profiling Interactive Analysis (GEPIA) box plots of S100A9 expression in TNBC, HER2 positive, Luminal-A and Luminal-B breast tumor subtypes. * indicates *p*-value Cutoff of 0.01.

**Additional file 4: Supplementary Fig. S4.** S100A8 and S100A9 expression levels in BC subtypes querying GOBO database. (A) Gene Set Analysis of S100A8 expression levels by GOBO dataset in Basal, HER2 positive, Luminal-A, Luminal-B, Normal-like and Unclassified BC subtypes. *p = < 0.00001*. (B) Gene Set Analysis of S100A9 expression levels by GOBO dataset in Basal, HER2 positive, Luminal-A, Luminal-B, Normal-like and Unclassified BC subtypes. *p = < 0.00001.* (C) Gene Set Analysis of combined S100A8 and S100A9 expression levels by GOBO dataset in Basal, HER2 positive, Luminal-A, Luminal-B, Normal-like and Unclassified BC subtypes. *p = < 0.00001.*

**Additional file 5: Supplementary Fig. S5.** RAGE expression correlates with a worse overall survival tendency in BC patients. (A) Overall survival in BC exhibiting high RAGE expression, as evaluated by a meta-analysis including 17 BC datasets. (B) Random effect model in BC exhibiting high RAGE expression. The value of each parameter is indicated within the box.

**Additional file 6: Supplementary Fig. S6.** S100A8/A9-RAGE system induces FAK activation in TNBC cells overexpressing RAGE. (A) pY397FAK and FAK immunoblots in MDA-MB231 cells overexpressing RAGE, transfected with siRNA targeting RAGE and treated with rhS100A8/A9 (100ng/ml) for 30 minutes. Knockdown efficiency of RAGE expression is shown. (B) pY397FAK and FAK immunoblots in BT-549 cells overexpressing RAGE, transfected with siRNA targeting RAGE and treated with rhS100A8/A9 (100ng/ml) for 30 minutes. Knockdown efficiency of RAGE expression is shown. (C) pY397FAK and FAK immunoblots in BT-549 cells overexpressing RAGE and treated for 30 minutes with rhS100A8/A9 (100ng/ml) alone or in combination with 1 μM RAGE antagonist FPS-ZM1. (D) pY397FAK and FAK immunoblots in BT-549 cells overexpressing RAGE and treated for 30 minutes with rhS100A8/A9 (100ng/ml) alone or in combination with 1 μM FAK inhibitor VS-4718. (E) pY397FAK and FAK immunoblots in BT-549 cells overexpressing RAGE and treated for 30 minutes with rhS100A8/A9 (100ng/ml) alone or in combination with 1 μM ROCK inhibitor Y-27632. (F) Cartoon depicting the proposed molecular mechanisms regulating FAK activation by S100A8/A9-RAGE system in TNBC cells. In immunoblotting assays ꞵ-actin served as loading control. Results shown are representative of three independent experiments performed in triplicate.

**Additional file 7: Supplementary Fig. S7.** Enriched pathways and related genes analysis in BC cohort expressing high RAGE levels. (A) Schematic representation of the putative significant pathways and their associated TFs predominantly enriched in BC cohort expressing high RAGE levels (*Z-score > 1*). (B) List of significantly up-regulated genes by TEAD2 and TEAD4 Hippo TFs in BC group expressing high RAGE levels (*Z-score > 1*). (C) Gene Expression Profiling Interactive Analysis (GEPIA) box plot of FLNB expression in BC subtypes. (D) Gene Expression Profiling Interactive Analysis (GEPIA) box plot of FLNC expression in BC subtypes.

**Additional file 8: Supplementary Fig. S8.** FLNA expression levels correlate with a worse overall survival tendency in BC patients. (A) Overall survival in BC tumors exhibiting high FLNA expression, as evaluated by a meta-analysis including 18 BC datasets. (B) Random effect model in BC tumors exhibiting high FLNA expression levels. The value of each parameter is indicated within the box.

**Additional file 9: Supplementary Fig. S9.** S100A8/A9-RAGE-FAK axis activates YAP in TNBC cells overexpressing RAGE. (A) pS127YAP and YAP immunoblots in BT-549 cells overexpressing RAGE and treated with rhS100A8/A9 (100ng/ml) for 60 minutes alone or in combination with 1 μM RAGE antagonist FPS-ZM1. (B) pS127YAP and YAP immunoblots in BT-549 cells overexpressing RAGE and treated with rhS100A8/A9 (100ng/ml) for 60 minutes alone or in combination with 1 μM FAK inhibitor VS-4718. (C) pS127YAP and YAP immunoblots in MDA-MB231 and BT-549 TNBC cells overexpressing RAGE, transfected with siRNA targeting RAGE and treated with rhS100A8/A9 (100ng/ml) for 60 minutes. Knockdown efficiency of RAGE expression is shown. (D) pS127YAP and YAP immunoblots in MDA-MB231 and BT-549 TNBC cells overexpressing RAGE, transfected with siRNA targeting FAK and treated with rhS100A8/A9 (100ng/ml) for 60 minutes. Knockdown efficiency of FAK expression is shown. In immunoblotting assays ꞵ-actin served as loading control. Results shown are representative of three independent experiments performed in triplicate.

**Additional file 10: Supplementary Fig. S10.** S100A8/A9-RAGE system regulates the expression of canonical Hippo/YAP target genes. (A) CTGF and Cyr61 mRNA levels in MDA-MB231 cells overexpressing RAGE and treated for 6 hours with rhS100A8/A9 (100ng/ml) alone or in combination with 1 μM RAGE antagonist FPS-ZM1. (B) CTGF and Cyr61 immunoblots in MDA-MB231 cells overexpressing RAGE and treated for 6 hours with rhS100A8/A9 (100ng/ml) alone or in combination with 1 μM RAGE antagonist FPS-ZM1. (C) CTGF and Cyr61 mRNA levels in MDA-MB231 cells overexpressing RAGE and treated for 6 hours with rhS100A8/A9 (100ng/ml) alone or in combination with 1 μM FAK inhibitor VS-4718. (D) CTGF and Cyr61 immunoblots in MDA-MB231 cells overexpressing RAGE and treated for 6 hours with rhS100A8/A9 (100ng/ml) alone or in combination with 1 μM FAK inhibitor VS-4718. Error bars represent mean ± SD. * indicates *p-value* < 0.05. In immunoblotting assays ꞵ-actin served as loading control. Results shown are representative of three independent experiments performed in triplicate.

**Additional file 11: Supplementary Fig. S11.** The inhibition of RAGE-FAK-YAP axis prevents S100A8/A9-mediated TNBC cell proliferation. (A) Proliferation of MDA-MB231 cells overexpressing RAGE, transfected with siRNA targeting RAGE, FAK and YAP, and then treated for 72 hours with 100 ng/ml rhS100A8/A9. (B) Knockdown efficiency of RAGE, FAK and YAP expression in MDA-MB231 cells overexpressing RAGE. (C) Proliferation of BT-549 cells overexpressing RAGE, transfected with siRNA targeting RAGE, FAK and YAP, and then treated for 72 hours with 100 ng/ml rhS100A8/A9. (D) Knockdown efficiency of RAGE, FAK and YAP expression in BT-549 cells overexpressing RAGE. Error bars represent mean ± SD. * indicates *p-value* < 0.05. In immunoblotting assays ꞵ-actin served as loading control. Results shown are representative of three independent experiments performed in triplicate.

## Data Availability

All data that were generated or analyzed during our study have been included in this article. Materials, additional data and protocols described within the manuscript will be made available from the authors upon reasonable request.

## Abbreviations

TNBC: Triple-Negative Breast Cancer
BC: Breast Cancer
ER: Estrogen Receptor
PR: Progesterone Receptor
Her-2: Human Epidermal Growth Factor Receptor-2
ICIs: Immune Checkpoint Inhibitors
DAMP: Damage-Associated Molecular Pattern
RAGE: Receptor for Advanced Glycation End-products
EMT: Epithelial-Mesenchymal Transition
FAK: Focal Adhesion Kinase
YAP: Yes-Associated Protein
CAF: Cancer-Associated Fibroblast
OS: Overall Survival
RFS: Relapse Free Survival
GEPIA2: Gene Expression Profiling Interactive Analysis 2
METABRIC: Molecular Taxonomy of Breast Cancer International Consortium
GSEA: Gene Set Enrichment Analysis
WebGestalt: WEB-based Gene SeT AnaLysis
FAs: Focal Adhesions
DEGs: Differentially Expressed Genes
TFs: Transcriptional Factors
FLNA: Filamin-A
AGEs: Advanced Glycation End Products
HMGB-1: High Mobility Group Box-1
