# Supplementary figures and images for: Focal Adhesion Kinase (FAK)-Hippo/YAP transduction signaling mediates the stimulatory effects exerted by S100A8/A9-RAGE system in triple-negative breast cancer (TNBC)
Source: J Exp Clin Cancer Res. 2022 Jun 3;41:193. doi: 10.1186/s13046-022-02396-0 (PMC9164429; doi:10.1186/s13046-022-02396-0)

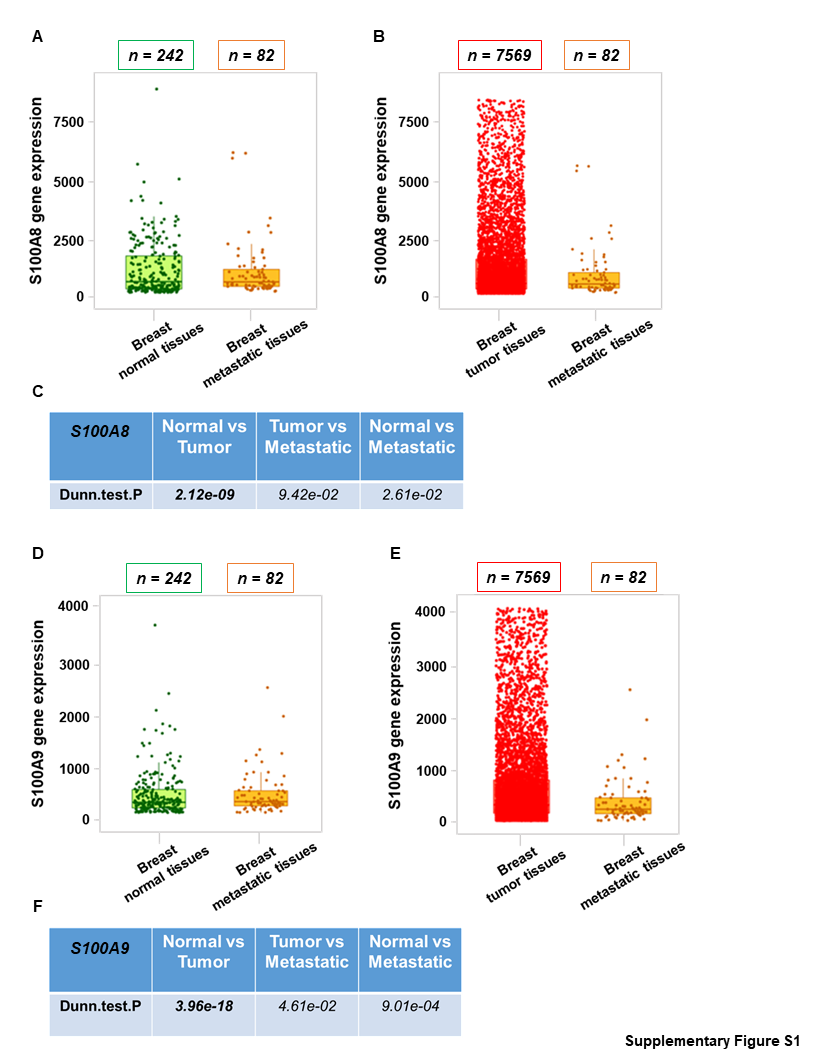

Supplement: Supplementary file 1 — Additional file 1: Supplementary Fig. S1. Expression of S100A8 and S100A9 in metastatic BC. (A) TNM box plot of S100A8 gene expression in normal (n = 242) and metastatic (n = 82) breast tissues. (B) TNM box plot of S100A8 gene expression in tumor (n = 7569) and metastatic (n = 82) breast tissues. (C) S100A8 expression levels in normal vs tumor, tumor vs metastatic, normal vs metastatic breast tissues, as evaluated by the Mann-Whitney U test. p-value is indicated within the box. (D) TNM box plot of S100A9 gene expression in normal (n = 242) and metastatic (n = 82) breast tissues. (E) TNM box plot of S100A9 gene expression in tumor (n = 7569) and metastatic (n = 82) breast tissues. (F) S100A9 expression levels in normal vs tumor, tumor vs metastatic, normal vs metastatic breast tissues, as evaluated by the Mann-Whitney U test. p-value is indicated within the box. [file 13046_2022_2396_MOESM1_ESM.tif]

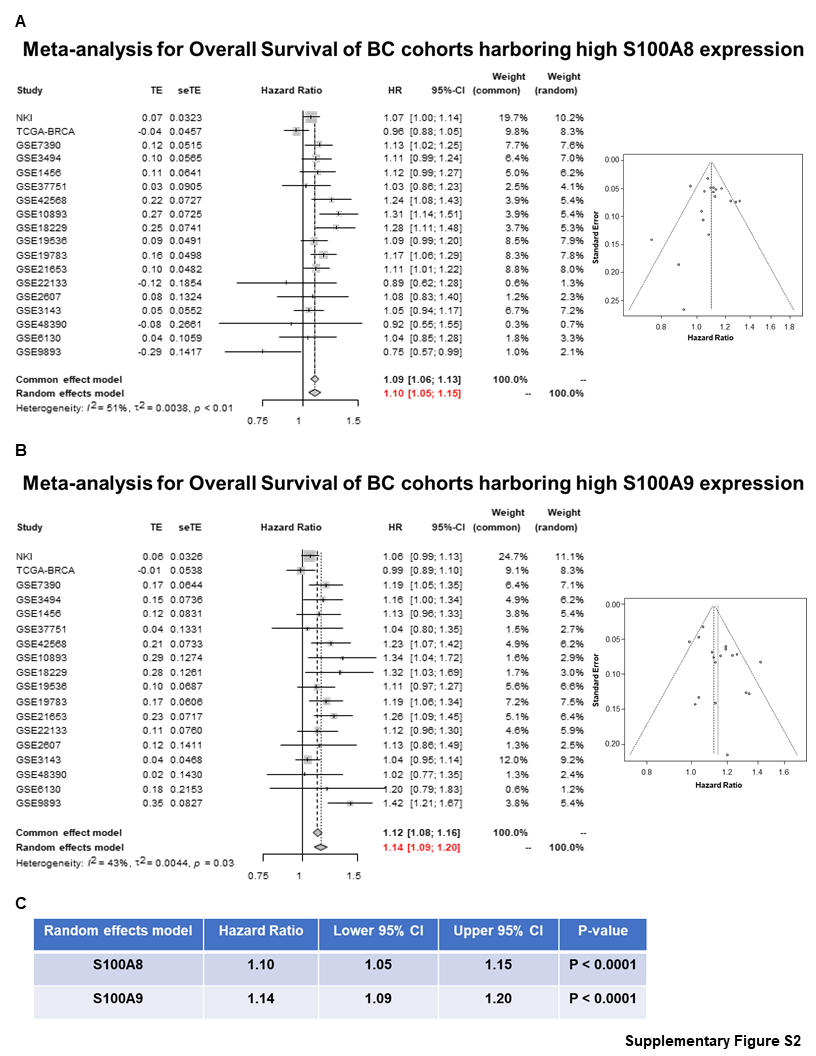

Supplement: Supplementary file 2 — Additional file 2: Supplementary Fig. S2. Expression levels of S100A8 and S100A9 correlate with a worse overall survival tendency in BC patients. (A) Evaluation of overall survival in BC tumors exhibiting high S100A8 expression, as evaluated by a meta-analysis including 18 BC datasets. (B) Evaluation of overall survival in BC tumors exhibiting high S100A9 expression, as evaluated by a meta-analysis including 18 BC datasets. (C) Random effect model in BC tumors exhibiting high S100A8 and S100A9 expression levels. The value of each parameter is indicated within the box. [file 13046_2022_2396_MOESM2_ESM.tif]

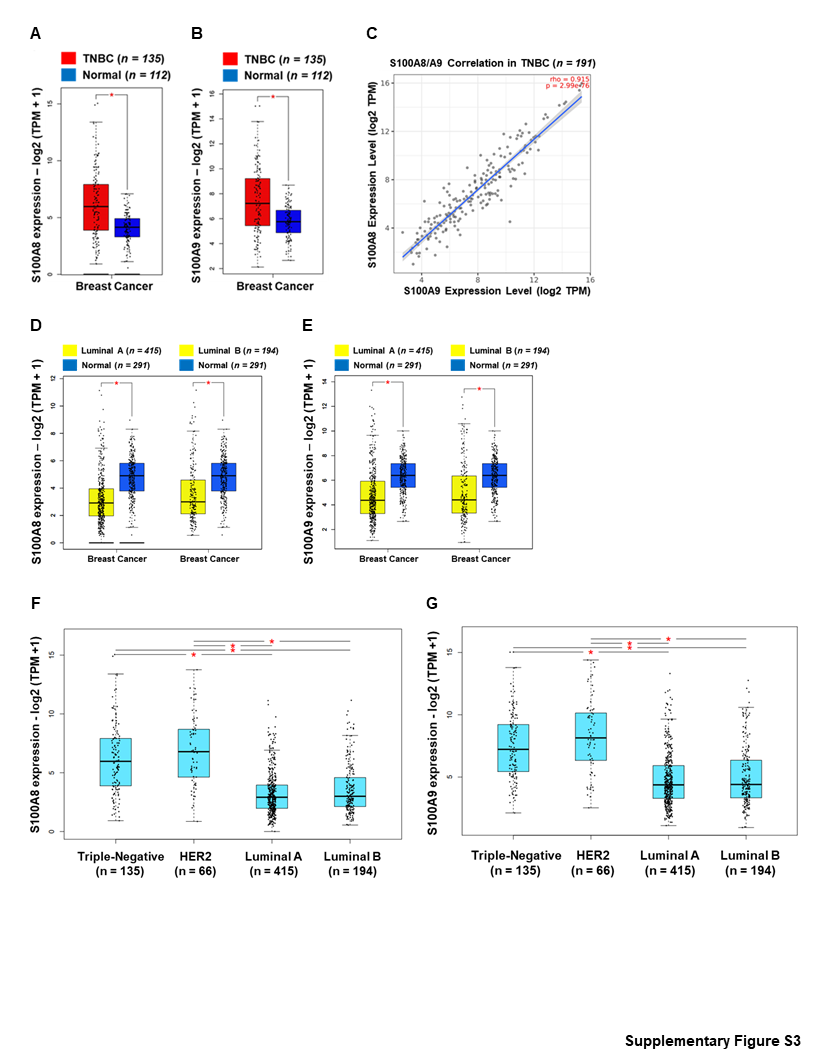

Supplement: Supplementary file 3 — Additional file 3: Supplementary Fig. S3. S100A8 and S100A9 expression levels in the different subtypes of BC. (A) Gene Expression Profiling Interactive Analysis (GEPIA) box plot of S100A8 expression in TNBC samples respect to normal breast samples. * indicates p-value Cutoff of 0.01. (B) Gene Expression Profiling Interactive Analysis (GEPIA) box plot of S100A9 expression in TNBC samples respect to normal breast samples. * indicates p-value Cutoff of 0.01. (C) Correlation between S100A8 and S100A9 expression levels in TNBC. (D) Gene Expression Profiling Interactive Analysis (GEPIA) box plots of S100A8 expression in Luminal-A samples respect to normal breast samples, and Luminal-B samples respect to normal breast samples. * indicates p-value Cutoff of 0.01. (E) Gene Expression Profiling Interactive Analysis (GEPIA) box plots of S100A9 expression in Luminal-A samples respect to normal breast samples, and Luminal-B samples respect to normal breast samples. * indicates p-value Cutoff of 0.01. (F) Gene Expression Profiling Interactive Analysis (GEPIA) box plots of S100A8 expression in TNBC, HER2 positive, Luminal-A and Luminal-B breast tumor subtypes. * indicates p-value Cutoff of 0.01. (G) Gene Expression Profiling Interactive Analysis (GEPIA) box plots of S100A9 expression in TNBC, HER2 positive, Luminal-A and Luminal-B breast tumor subtypes. * indicates p-value Cutoff of 0.01. [file 13046_2022_2396_MOESM3_ESM.tif]

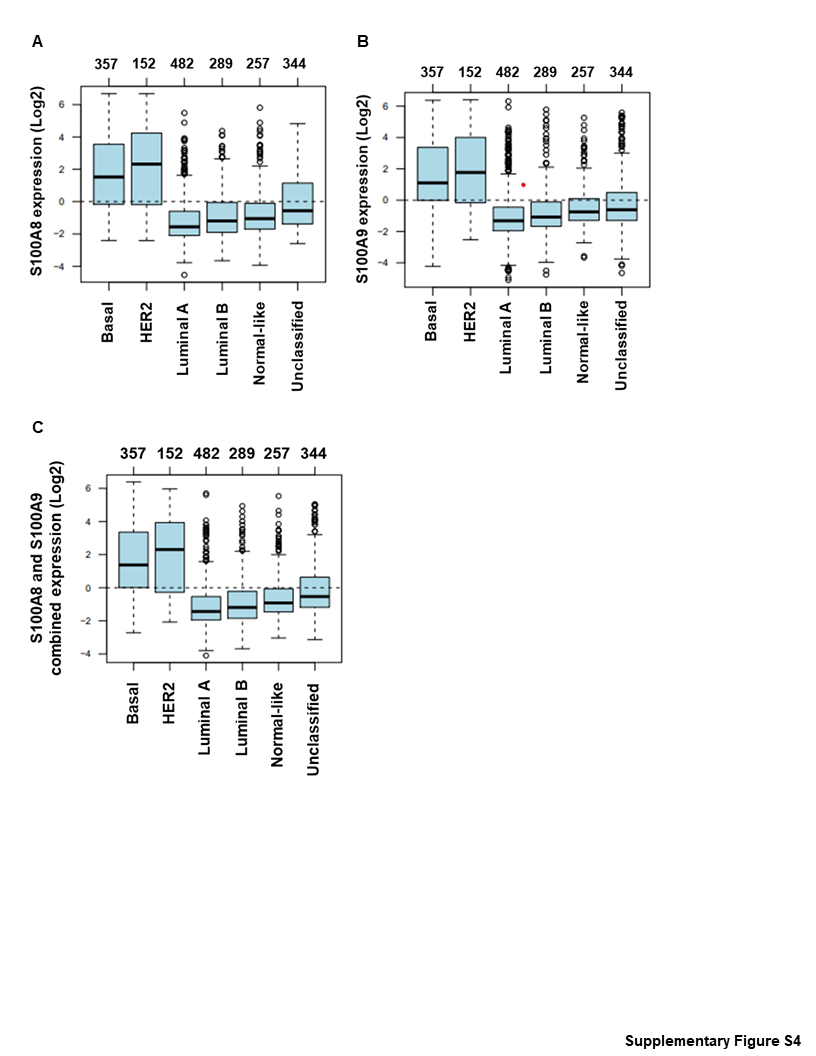

Supplement: Supplementary file 4 — Additional file 4: Supplementary Fig. S4. S100A8 and S100A9 expression levels in BC subtypes querying GOBO database. (A) Gene Set Analysis of S100A8 expression levels by GOBO dataset in Basal, HER2 positive, Luminal-A, Luminal-B, Normal-like and Unclassified BC subtypes. p = < 0.00001. (B) Gene Set Analysis of S100A9 expression levels by GOBO dataset in Basal, HER2 positive, Luminal-A, Luminal-B, Normal-like and Unclassified BC subtypes. p = < 0.00001. (C) Gene Set Analysis of combined S100A8 and S100A9 expression levels by GOBO dataset in Basal, HER2 positive, Luminal-A, Luminal-B, Normal-like and Unclassified BC subtypes. p = < 0.00001. [file 13046_2022_2396_MOESM4_ESM.tif]

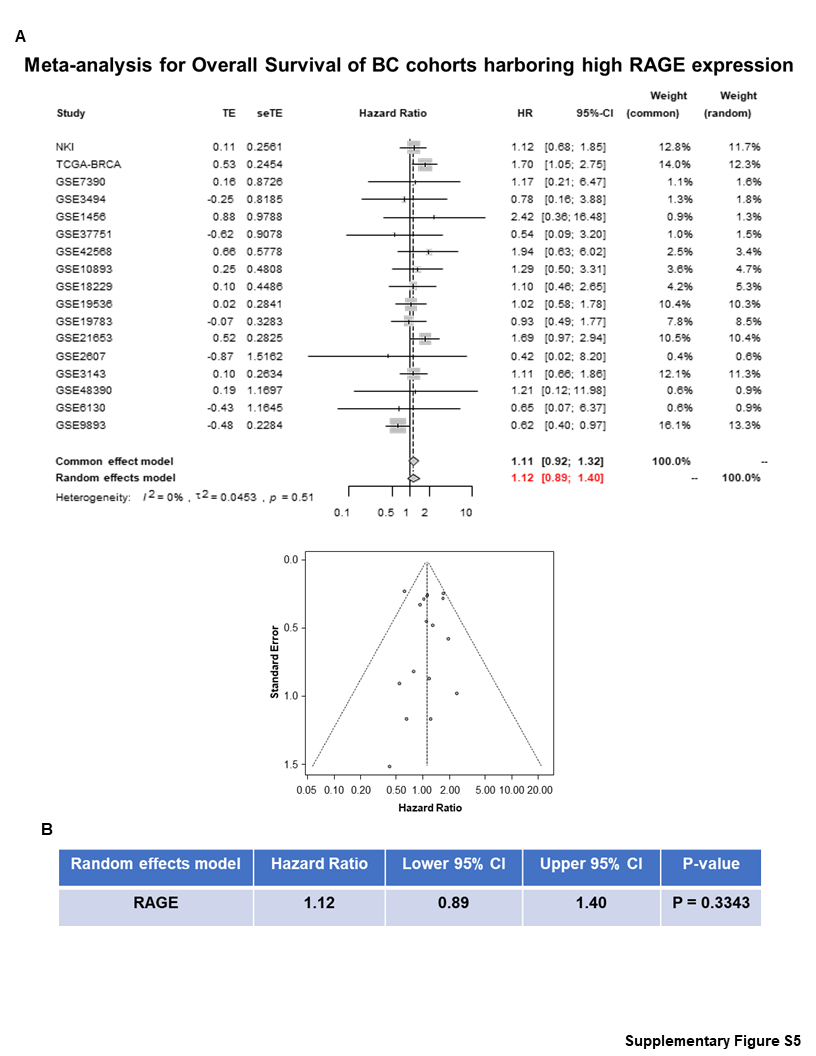

Supplement: Supplementary file 5 — Additional file 5: Supplementary Fig. S5. RAGE expression correlates with a worse overall survival tendency in BC patients. (A) Overall survival in BC exhibiting high RAGE expression, as evaluated by a meta-analysis including 17 BC datasets. (B) Random effect model in BC exhibiting high RAGE expression. The value of each parameter is indicated within the box. [file 13046_2022_2396_MOESM5_ESM.tif]

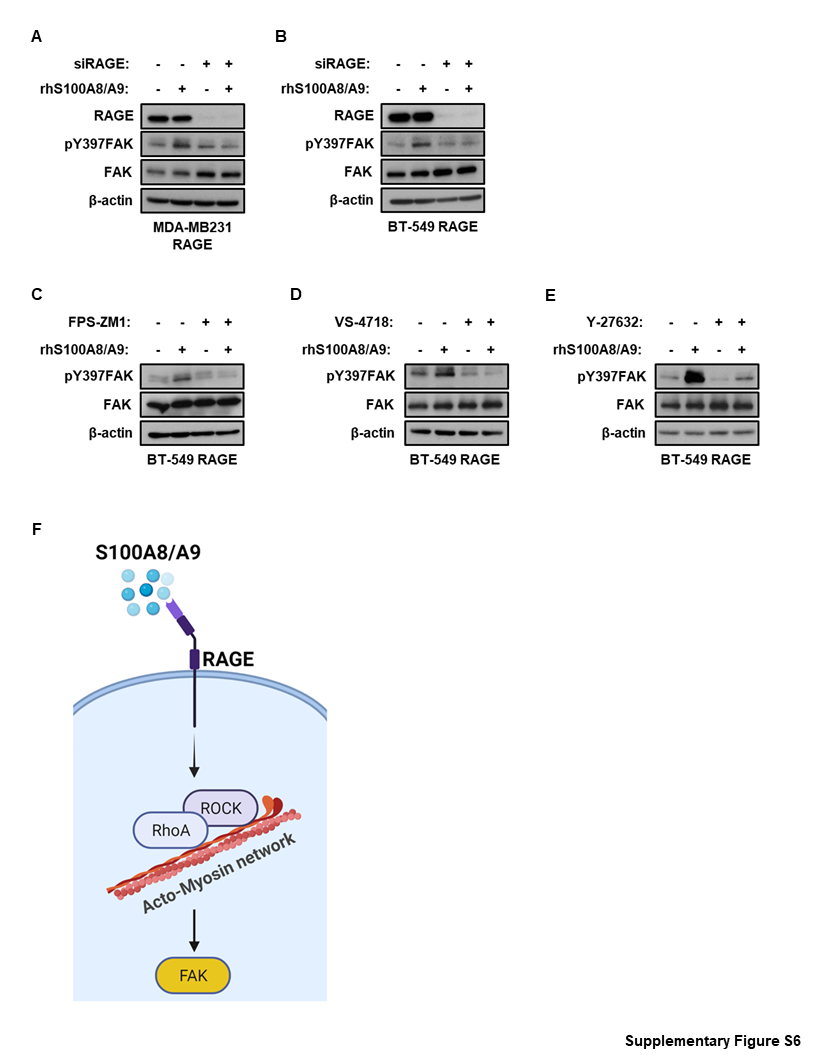

Supplement: Supplementary file 6 — Additional file 6: Supplementary Fig. S6. S100A8/A9-RAGE system induces FAK activation in TNBC cells overexpressing RAGE. (A) pY397FAK and FAK immunoblots in MDA-MB231 cells overexpressing RAGE, transfected with siRNA targeting RAGE and treated with rhS100A8/A9 (100ng/ml) for 30 minutes. Knockdown efficiency of RAGE expression is shown. (B) pY397FAK and FAK immunoblots in BT-549 cells overexpressing RAGE, transfected with siRNA targeting RAGE and treated with rhS100A8/A9 (100ng/ml) for 30 minutes. Knockdown efficiency of RAGE expression is shown. (C) pY397FAK and FAK immunoblots in BT-549 cells overexpressing RAGE and treated for 30 minutes with rhS100A8/A9 (100ng/ml) alone or in combination with 1 μM RAGE antagonist FPS-ZM1. (D) pY397FAK and FAK immunoblots in BT-549 cells overexpressing RAGE and treated for 30 minutes with rhS100A8/A9 (100ng/ml) alone or in combination with 1 μM FAK inhibitor VS-4718. (E) pY397FAK and FAK immunoblots in BT-549 cells overexpressing RAGE and treated for 30 minutes with rhS100A8/A9 (100ng/ml) alone or in combination with 1 μM ROCK inhibitor Y-27632. (F) Cartoon depicting the proposed molecular mechanisms regulating FAK activation by S100A8/A9-RAGE system in TNBC cells. In immunoblotting assays ꞵ-actin served as loading control. Results shown are representative of three independent experiments performed in triplicate. [file 13046_2022_2396_MOESM6_ESM.tif]

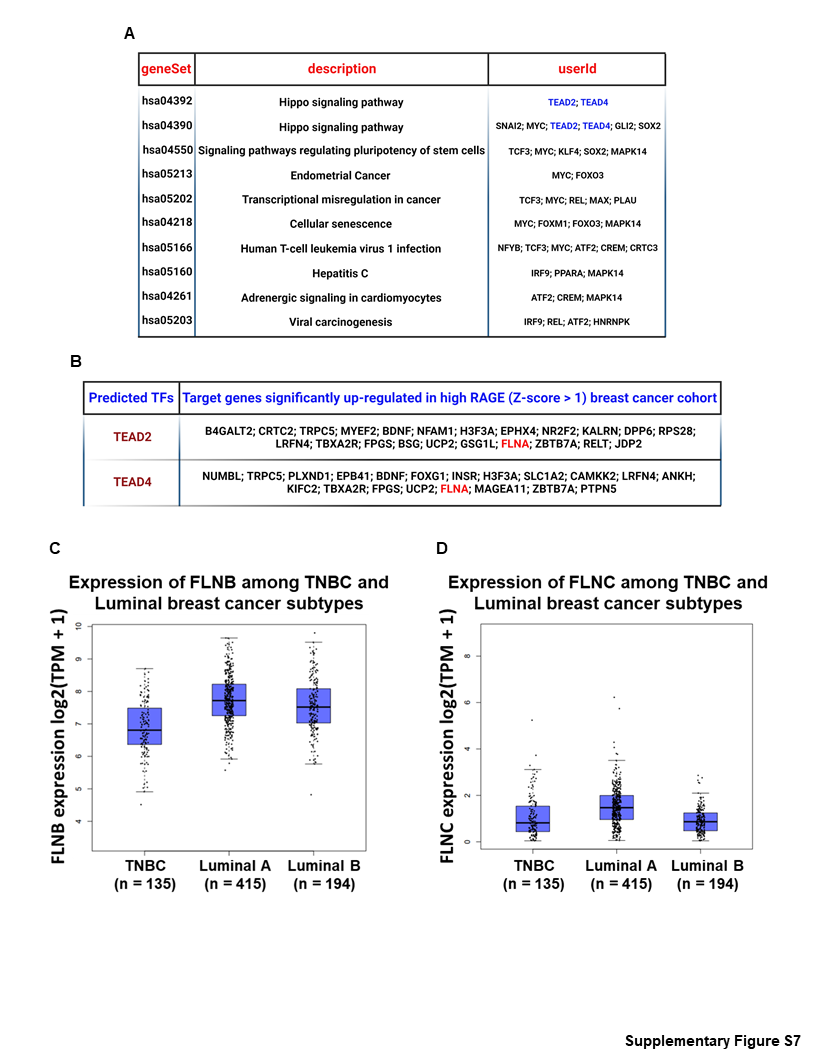

Supplement: Supplementary file 7 — Additional file 7: Supplementary Fig. S7. Enriched pathways and related genes analysis in BC cohort expressing high RAGE levels. (A) Schematic representation of the putative significant pathways and their associated TFs predominantly enriched in BC cohort expressing high RAGE levels (Z-score > 1). (B) List of significantly up-regulated genes by TEAD2 and TEAD4 Hippo TFs in BC group expressing high RAGE levels (Z-score > 1). (C) Gene Expression Profiling Interactive Analysis (GEPIA) box plot of FLNB expression in BC subtypes. (D) Gene Expression Profiling Interactive Analysis (GEPIA) box plot of FLNC expression in BC subtypes. [file 13046_2022_2396_MOESM7_ESM.tif]

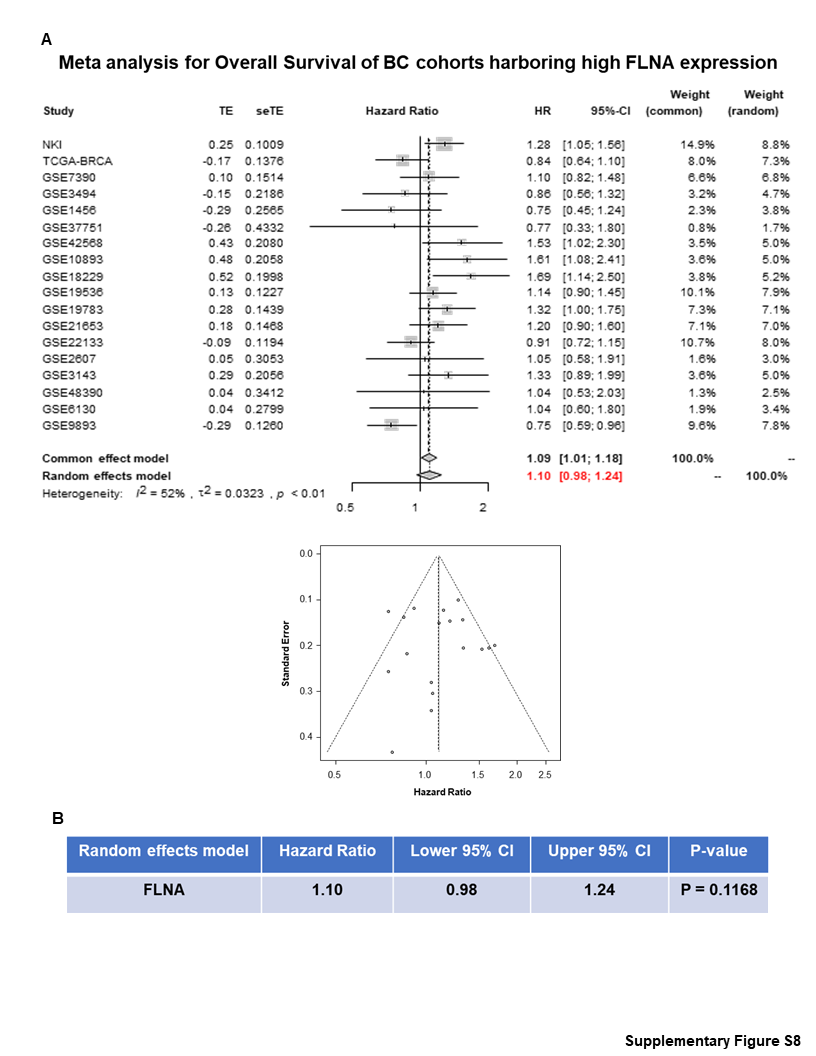

Supplement: Supplementary file 8 — Additional file 8: Supplementary Fig. S8. FLNA expression levels correlate with a worse overall survival tendency in BC patients. (A) Overall survival in BC tumors exhibiting high FLNA expression, as evaluated by a meta-analysis including 18 BC datasets. (B) Random effect model in BC tumors exhibiting high FLNA expression levels. The value of each parameter is indicated within the box. [file 13046_2022_2396_MOESM8_ESM.tif]

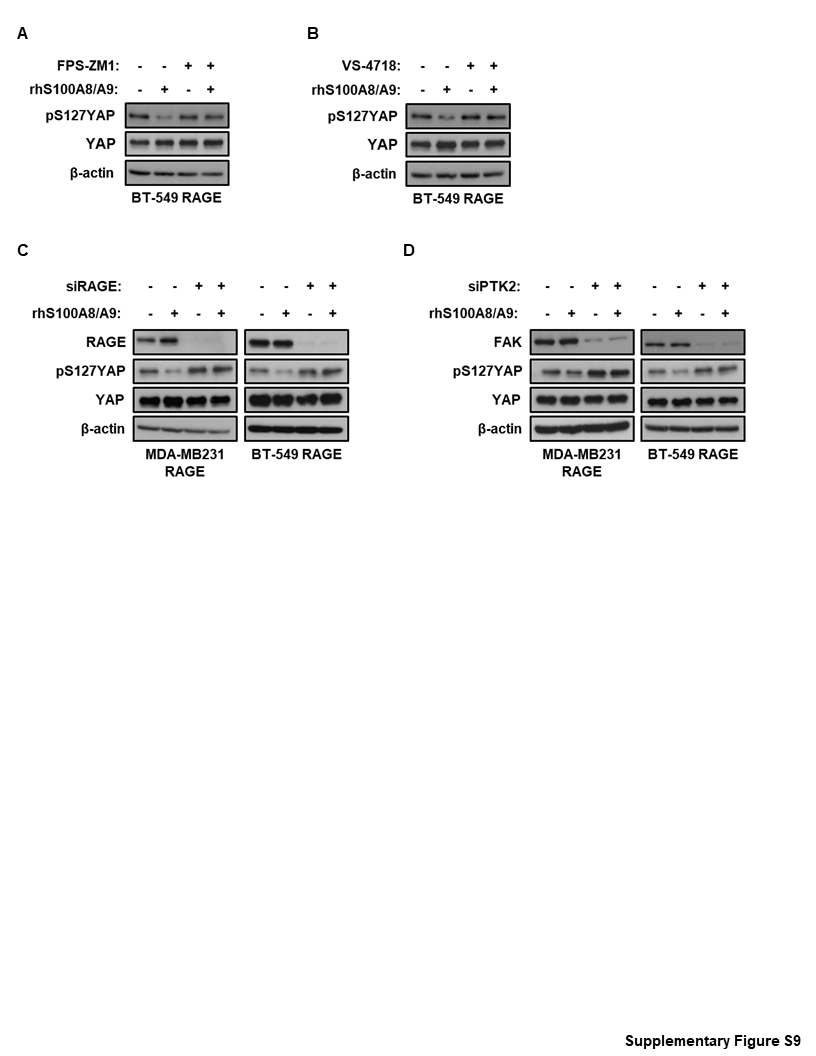

Supplement: Supplementary file 9 — Additional file 9: Supplementary Fig. S9. S100A8/A9-RAGE-FAK axis activates YAP in TNBC cells overexpressing RAGE. (A) pS127YAP and YAP immunoblots in BT-549 cells overexpressing RAGE and treated with rhS100A8/A9 (100ng/ml) for 60 minutes alone or in combination with 1 μM RAGE antagonist FPS-ZM1. (B) pS127YAP and YAP immunoblots in BT-549 cells overexpressing RAGE and treated with rhS100A8/A9 (100ng/ml) for 60 minutes alone or in combination with 1 μM FAK inhibitor VS-4718. (C) pS127YAP and YAP immunoblots in MDA-MB231 and BT-549 TNBC cells overexpressing RAGE, transfected with siRNA targeting RAGE and treated with rhS100A8/A9 (100ng/ml) for 60 minutes. Knockdown efficiency of RAGE expression is shown. (D) pS127YAP and YAP immunoblots in MDA-MB231 and BT-549 TNBC cells overexpressing RAGE, transfected with siRNA targeting FAK and treated with rhS100A8/A9 (100ng/ml) for 60 minutes. Knockdown efficiency of FAK expression is shown. In immunoblotting assays ꞵ-actin served as loading control. Results shown are representative of three independent experiments performed in triplicate. [file 13046_2022_2396_MOESM9_ESM.tif]

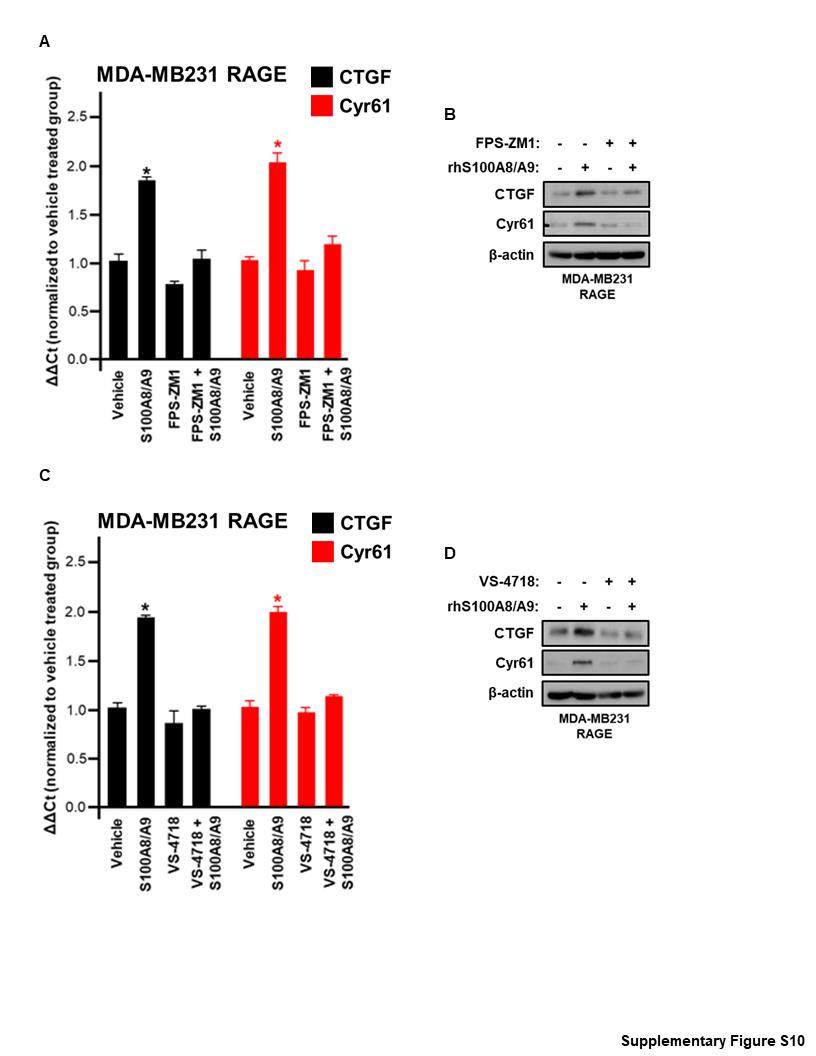

Supplement: Supplementary file 10 — Additional file 10: Supplementary Fig. S10. S100A8/A9-RAGE system regulates the expression of canonical Hippo/YAP target genes. (A) CTGF and Cyr61 mRNA levels in MDA-MB231 cells overexpressing RAGE and treated for 6 hours with rhS100A8/A9 (100ng/ml) alone or in combination with 1 μM RAGE antagonist FPS-ZM1. (B) CTGF and Cyr61 immunoblots in MDA-MB231 cells overexpressing RAGE and treated for 6 hours with rhS100A8/A9 (100ng/ml) alone or in combination with 1 μM RAGE antagonist FPS-ZM1. (C) CTGF and Cyr61 mRNA levels in MDA-MB231 cells overexpressing RAGE and treated for 6 hours with rhS100A8/A9 (100ng/ml) alone or in combination with 1 μM FAK inhibitor VS-4718. (D) CTGF and Cyr61 immunoblots in MDA-MB231 cells overexpressing RAGE and treated for 6 hours with rhS100A8/A9 (100ng/ml) alone or in combination with 1 μM FAK inhibitor VS-4718. Error bars represent mean ± SD. * indicates p-value < 0.05. In immunoblotting assays ꞵ-actin served as loading control. Results shown are representative of three independent experiments performed in triplicate. [file 13046_2022_2396_MOESM10_ESM.tif]

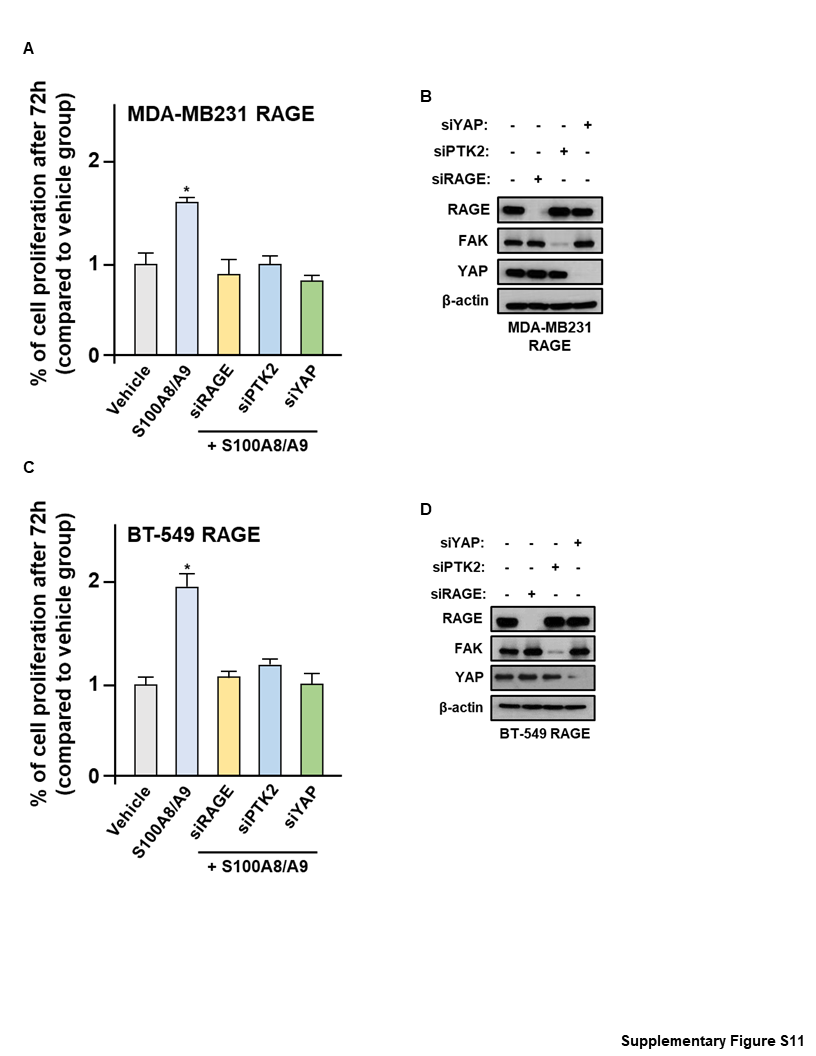

Supplement: Supplementary file 11 — Additional file 11: Supplementary Fig. S11. The inhibition of RAGE-FAK-YAP axis prevents S100A8/A9-mediated TNBC cell proliferation. (A) Proliferation of MDA-MB231 cells overexpressing RAGE, transfected with siRNA targeting RAGE, FAK and YAP, and then treated for 72 hours with 100 ng/ml rhS100A8/A9. (B) Knockdown efficiency of RAGE, FAK and YAP expression in MDA-MB231 cells overexpressing RAGE. (C) Proliferation of BT-549 cells overexpressing RAGE, transfected with siRNA targeting RAGE, FAK and YAP, and then treated for 72 hours with 100 ng/ml rhS100A8/A9. (D) Knockdown efficiency of RAGE, FAK and YAP expression in BT-549 cells overexpressing RAGE. Error bars represent mean ± SD. * indicates p-value < 0.05. In immunoblotting assays ꞵ-actin served as loading control. Results shown are representative of three independent experiments performed in triplicate. [file 13046_2022_2396_MOESM11_ESM.tif]
